# Search for direct pair production of a chargino and a neutralino decaying to the 125 GeV Higgs boson in $\sqrt{\varvec{s}} = 8$ TeV $\varvec{pp}$ collisions with the ATLAS detector

**DOI:** 10.1140/epjc/s10052-015-3408-7

**Published:** 2015-05-12

**Authors:** G. Aad, B. Abbott, J. Abdallah, S. Abdel Khalek, O. Abdinov, R. Aben, B. Abi, M. Abolins, O. S. AbouZeid, H. Abramowicz, H. Abreu, R. Abreu, Y. Abulaiti, B. S. Acharya, L. Adamczyk, D. L. Adams, J. Adelman, S. Adomeit, T. Adye, T. Agatonovic-Jovin, J. A. Aguilar-Saavedra, M. Agustoni, S. P. Ahlen, F. Ahmadov, G. Aielli, H. Akerstedt, T. P. A. Åkesson, G. Akimoto, A. V. Akimov, G. L. Alberghi, J. Albert, S. Albrand, M. J. Alconada Verzini, M. Aleksa, I. N. Aleksandrov, C. Alexa, G. Alexander, G. Alexandre, T. Alexopoulos, M. Alhroob, G. Alimonti, L. Alio, J. Alison, B. M. M. Allbrooke, L. J. Allison, P. P. Allport, A. Aloisio, A. Alonso, F. Alonso, C. Alpigiani, A. Altheimer, B. Alvarez Gonzalez, M. G. Alviggi, K. Amako, Y. Amaral Coutinho, C. Amelung, D. Amidei, S. P. Amor Dos Santos, A. Amorim, S. Amoroso, N. Amram, G. Amundsen, C. Anastopoulos, L. S. Ancu, N. Andari, T. Andeen, C. F. Anders, G. Anders, K. J. Anderson, A. Andreazza, V. Andrei, X. S. Anduaga, S. Angelidakis, I. Angelozzi, P. Anger, A. Angerami, F. Anghinolfi, A. V. Anisenkov, N. Anjos, A. Annovi, M. Antonelli, A. Antonov, J. Antos, F. Anulli, M. Aoki, L. Aperio Bella, G. Arabidze, Y. Arai, J. P. Araque, A. T. H. Arce, F. A Arduh, J.-F. Arguin, S. Argyropoulos, M. Arik, A. J. Armbruster, O. Arnaez, V. Arnal, H. Arnold, M. Arratia, O. Arslan, A. Artamonov, G. Artoni, S. Asai, N. Asbah, A. Ashkenazi, B. Åsman, L. Asquith, K. Assamagan, R. Astalos, M. Atkinson, N. B. Atlay, B. Auerbach, K. Augsten, M. Aurousseau, G. Avolio, B. Axen, M. K. Ayoub, G. Azuelos, M. A. Baak, A. E. Baas, C. Bacci, H. Bachacou, K. Bachas, M. Backes, M. Backhaus, P. Bagiacchi, P. Bagnaia, Y. Bai, T. Bain, J. T. Baines, O. K. Baker, P. Balek, T. Balestri, F. Balli, E. Banas, Sw. Banerjee, A. A. E. Bannoura, H. S. Bansil, L. Barak, S. P. Baranov, E. L. Barberio, D. Barberis, M. Barbero, T. Barillari, M. Barisonzi, T. Barklow, N. Barlow, S. L. Barnes, B. M. Barnett, R. M. Barnett, Z. Barnovska, A. Baroncelli, G. Barone, A. J. Barr, F. Barreiro, J. Barreiro Guimarães da Costa, R. Bartoldus, A. E. Barton, P. Bartos, A. Bassalat, A. Basye, R. L. Bates, S. J. Batista, J. R. Batley, M. Battaglia, M. Bauce, F. Bauer, H. S. Bawa, J. B. Beacham, M. D. Beattie, T. Beau, P. H. Beauchemin, R. Beccherle, P. Bechtle, H. P. Beck, K. Becker, S. Becker, M. Beckingham, C. Becot, A. J. Beddall, A. Beddall, V. A. Bednyakov, C. P. Bee, L. J. Beemster, T. A. Beermann, M. Begel, K. Behr, C. Belanger-Champagne, P. J. Bell, W. H. Bell, G. Bella, L. Bellagamba, A. Bellerive, M. Bellomo, K. Belotskiy, O. Beltramello, O. Benary, D. Benchekroun, M. Bender, K. Bendtz, N. Benekos, Y. Benhammou, E. Benhar Noccioli, J. A. Benitez Garcia, D. P. Benjamin, J. R. Bensinger, S. Bentvelsen, L. Beresford, M. Beretta, D. Berge, E. Bergeaas Kuutmann, N. Berger, F. Berghaus, J. Beringer, C. Bernard, N. R. Bernard, C. Bernius, F. U. Bernlochner, T. Berry, P. Berta, C. Bertella, G. Bertoli, F. Bertolucci, C. Bertsche, D. Bertsche, M. I. Besana, G. J. Besjes, O. Bessidskaia Bylund, M. Bessner, N. Besson, C. Betancourt, S. Bethke, A. J. Beven, W. Bhimji, R. M. Bianchi, L. Bianchini, M. Bianco, O. Biebel, S. P. Bieniek, M. Biglietti, J. Bilbao De Mendizabal, H. Bilokon, M. Bindi, S. Binet, A. Bingul, C. Bini, C. W. Black, J. E. Black, K. M. Black, D. Blackburn, R. E. Blair, J.-B. Blanchard, J.E. Blanco, T. Blazek, I. Bloch, C. Blocker, W. Blum, U. Blumenschein, G. J. Bobbink, V. S. Bobrovnikov, S. S. Bocchetta, A. Bocci, C. Bock, C. R. Boddy, M. Boehler, J. A. Bogaerts, A. G. Bogdanchikov, C. Bohm, V. Boisvert, T. Bold, V. Boldea, A. S. Boldyrev, M. Bomben, M. Bona, M. Boonekamp, A. Borisov, G. Borissov, S. Borroni, J. Bortfeldt, V. Bortolotto, K. Bos, D. Boscherini, M. Bosman, J. Boudreau, J. Bouffard, E. V. Bouhova-Thacker, D. Boumediene, C. Bourdarios, N. Bousson, S. Boutouil, A. Boveia, J. Boyd, I. R. Boyko, I. Bozic, J. Bracinik, A. Brandt, G. Brandt, O. Brandt, U. Bratzler, B. Brau, J. E. Brau, H. M. Braun, S. F. Brazzale, K. Brendlinger, A. J. Brennan, L. Brenner, R. Brenner, S. Bressler, K. Bristow, T. M. Bristow, D. Britton, F. M. Brochu, I. Brock, R. Brock, J. Bronner, G. Brooijmans, T. Brooks, W. K. Brooks, J. Brosamer, E. Brost, J. Brown, P. A. Bruckman de Renstrom, D. Bruncko, R. Bruneliere, A. Bruni, G. Bruni, M. Bruschi, L. Bryngemark, T. Buanes, Q. Buat, F. Bucci, P. Buchholz, A. G. Buckley, S. I. Buda, I. A. Budagov, F. Buehrer, L. Bugge, M. K. Bugge, O. Bulekov, H. Burckhart, S. Burdin, B. Burghgrave, S. Burke, I. Burmeister, E. Busato, D. Büscher, V. Büscher, P. Bussey, C. P. Buszello, J. M. Butler, A. I. Butt, C. M. Buttar, J. M. Butterworth, P. Butti, W. Buttinger, A. Buzatu, S. Cabrera Urbán, D. Caforio, O. Cakir, P. Calafiura, A. Calandri, G. Calderini, P. Calfayan, L. P. Caloba, D. Calvet, S. Calvet, R. Camacho Toro, S. Camarda, D. Cameron, L. M. Caminada, R. Caminal Armadans, S. Campana, M. Campanelli, A. Campoverde, V. Canale, A. Canepa, M. Cano Bret, J. Cantero, R. Cantrill, T. Cao, M. D. M. Capeans Garrido, I. Caprini, M. Caprini, M. Capua, R. Caputo, R. Cardarelli, T. Carli, G. Carlino, L. Carminati, S. Caron, E. Carquin, G. D. Carrillo-Montoya, J. R. Carter, J. Carvalho, D. Casadei, M. P. Casado, M. Casolino, E. Castaneda-Miranda, A. Castelli, V. Castillo Gimenez, N. F. Castro, P. Catastini, A. Catinaccio, J. R. Catmore, A. Cattai, G. Cattani, J. Caudron, V. Cavaliere, D. Cavalli, M. Cavalli-Sforza, V. Cavasinni, F. Ceradini, B. C. Cerio, K. Cerny, A. S. Cerqueira, A. Cerri, L. Cerrito, F. Cerutti, M. Cerv, A. Cervelli, S. A. Cetin, A. Chafaq, D. Chakraborty, I. Chalupkova, P. Chang, B. Chapleau, J. D. Chapman, D. Charfeddine, D. G. Charlton, C. C. Chau, C. A. Chavez Barajas, S. Cheatham, A. Chegwidden, S. Chekanov, S. V. Chekulaev, G. A. Chelkov, M. A. Chelstowska, C. Chen, H. Chen, K. Chen, L. Chen, S. Chen, X. Chen, Y. Chen, H. C. Cheng, Y. Cheng, A. Cheplakov, E. Cheremushkina, R. Cherkaoui El Moursli, V. Chernyatin, E. Cheu, L. Chevalier, V. Chiarella, J. T. Childers, A. Chilingarov, G. Chiodini, A. S. Chisholm, R. T. Chislett, A. Chitan, M. V. Chizhov, S. Chouridou, B. K. B. Chow, D. Chromek-Burckhart, M. L. Chu, J. Chudoba, J. J. Chwastowski, L. Chytka, G. Ciapetti, A. K. Ciftci, D. Cinca, V. Cindro, A. Ciocio, Z. H. Citron, M. Citterio, M. Ciubancan, A. Clark, P. J. Clark, R. N. Clarke, W. Cleland, C. Clement, Y. Coadou, M. Cobal, A. Coccaro, J. Cochran, L. Coffey, J. G. Cogan, B. Cole, S. Cole, A. P. Colijn, J. Collot, T. Colombo, G. Compostella, P. Conde Muiño, E. Coniavitis, S. H. Connell, I. A. Connelly, S. M. Consonni, V. Consorti, S. Constantinescu, C. Conta, G. Conti, F. Conventi, M. Cooke, B. D. Cooper, A. M. Cooper-Sarkar, K. Copic, T. Cornelissen, M. Corradi, F. Corriveau, A. Corso-Radu, A. Cortes-Gonzalez, G. Cortiana, M. J. Costa, D. Costanzo, D. Côté, G. Cottin, G. Cowan, B. E. Cox, K. Cranmer, G. Cree, S. Crépé-Renaudin, F. Crescioli, W. A. Cribbs, M. Crispin Ortuzar, M. Cristinziani, V. Croft, G. Crosetti, T. Cuhadar Donszelmann, J. Cummings, M. Curatolo, C. Cuthbert, H. Czirr, P. Czodrowski, S. D’Auria, M. D’Onofrio, M. J. Da Cunha Sargedas De Sousa, C. Da Via, W. Dabrowski, A. Dafinca, T. Dai, O. Dale, F. Dallaire, C. Dallapiccola, M. Dam, J. R. Dandoy, A. C. Daniells, M. Danninger, M. Dano Hoffmann, V. Dao, G. Darbo, S. Darmora, J. Dassoulas, A. Dattagupta, W. Davey, C. David, T. Davidek, E. Davies, M. Davies, O. Davignon, P. Davison, Y. Davygora, E. Dawe, I. Dawson, R. K. Daya-Ishmukhametova, K. De, R. de Asmundis, S. De Castro, S. De Cecco, N. De Groot, P. de Jong, H. De la Torre, F. De Lorenzi, L. De Nooij, D. De Pedis, A. De Salvo, U. De Sanctis, A. De Santo, J. B. De Vivie De Regie, W. J. Dearnaley, R. Debbe, C. Debenedetti, D. V. Dedovich, I. Deigaard, J. Del Peso, T. Del Prete, D. Delgove, F. Deliot, C. M. Delitzsch, M. Deliyergiyev, A. Dell’Acqua, L. Dell’Asta, M. Dell’Orso, M. Della Pietra, D. della Volpe, M. Delmastro, P. A. Delsart, C. Deluca, D. A. DeMarco, S. Demers, M. Demichev, A. Demilly, S. P. Denisov, D. Derendarz, J. E. Derkaoui, F. Derue, P. Dervan, K. Desch, C. Deterre, P. O. Deviveiros, A. Dewhurst, S. Dhaliwal, A. Di Ciaccio, L. Di Ciaccio, A. Di Domenico, C. Di Donato, A. Di Girolamo, B. Di Girolamo, A. Di Mattia, B. Di Micco, R. Di Nardo, A. Di Simone, R. Di Sipio, D. Di Valentino, C. Diaconu, M. Diamond, F. A. Dias, M. A. Diaz, E. B. Diehl, J. Dietrich, T. A. Dietzsch, S. Diglio, A. Dimitrievska, J. Dingfelder, F. Dittus, F. Djama, T. Djobava, J. I. Djuvsland, M. A. B. do Vale, D. Dobos, M. Dobre, C. Doglioni, T. Doherty, T. Dohmae, J. Dolejsi, Z. Dolezal, B. A. Dolgoshein, M. Donadelli, S. Donati, P. Dondero, J. Donini, J. Dopke, A. Doria, M. T. Dova, A. T. Doyle, M. Dris, E. Dubreuil, E. Duchovni, G. Duckeck, O. A. Ducu, D. Duda, A. Dudarev, L. Duflot, L. Duguid, M. Dührssen, M. Dunford, H. Duran Yildiz, M. Düren, A. Durglishvili, D. Duschinger, M. Dwuznik, M. Dyndal, W. Edson, N. C. Edwards, W. Ehrenfeld, T. Eifert, G. Eigen, K. Einsweiler, T. Ekelof, M. El Kacimi, M. Ellert, S. Elles, F. Ellinghaus, A. A. Elliot, N. Ellis, J. Elmsheuser, M. Elsing, D. Emeliyanov, Y. Enari, O. C. Endner, M. Endo, R. Engelmann, J. Erdmann, A. Ereditato, D. Eriksson, G. Ernis, J. Ernst, M. Ernst, S. Errede, E. Ertel, M. Escalier, H. Esch, C. Escobar, B. Esposito, A. I. Etienvre, E. Etzion, H. Evans, A. Ezhilov, L. Fabbri, G. Facini, R. M. Fakhrutdinov, S. Falciano, R. J. Falla, J. Faltova, Y. Fang, M. Fanti, A. Farbin, A. Farilla, T. Farooque, S. Farrell, S. M. Farrington, P. Farthouat, F. Fassi, P. Fassnacht, D. Fassouliotis, A. Favareto, L. Fayard, P. Federic, O. L. Fedin, W. Fedorko, S. Feigl, L. Feligioni, C. Feng, E. J. Feng, H. Feng, A. B. Fenyuk, P. Fernandez Martinez, S. Fernandez Perez, S. Ferrag, J. Ferrando, A. Ferrari, P. Ferrari, R. Ferrari, D. E. Ferreira de Lima, A. Ferrer, D. Ferrere, C. Ferretti, A. Ferretto Parodi, M. Fiascaris, F. Fiedler, A. Filipčič, M. Filipuzzi, F. Filthaut, M. Fincke-Keeler, K. D. Finelli, M. C. N. Fiolhais, L. Fiorini, A. Firan, A. Fischer, J. Fischer, W. C. Fisher, E. A. Fitzgerald, M. Flechl, I. Fleck, P. Fleischmann, S. Fleischmann, G. T. Fletcher, G. Fletcher, T. Flick, A. Floderus, L. R. Flores Castillo, M. J. Flowerdew, A. Formica, A. Forti, D. Fournier, H. Fox, S. Fracchia, P. Francavilla, M. Franchini, D. Francis, L. Franconi, M. Franklin, M. Fraternali, D. Freeborn, S. T. French, F. Friedrich, D. Froidevaux, J. A. Frost, C. Fukunaga, E. Fullana Torregrosa, B. G. Fulsom, J. Fuster, C. Gabaldon, O. Gabizon, A. Gabrielli, A. Gabrielli, S. Gadatsch, S. Gadomski, G. Gagliardi, P. Gagnon, C. Galea, B. Galhardo, E. J. Gallas, B. J. Gallop, P. Gallus, G. Galster, K. K. Gan, J. Gao, Y. S. Gao, F. M. Garay Walls, F. Garberson, C. García, J. E. García Navarro, M. Garcia-Sciveres, R. W. Gardner, N. Garelli, V. Garonne, C. Gatti, G. Gaudio, B. Gaur, L. Gauthier, P. Gauzzi, I. L. Gavrilenko, C. Gay, G. Gaycken, E. N. Gazis, P. Ge, Z. Gecse, C. N. P. Gee, D. A. A. Geerts, Ch. Geich-Gimbel, C. Gemme, M. H. Genest, S. Gentile, M. George, S. George, D. Gerbaudo, A. Gershon, H. Ghazlane, N. Ghodbane, B. Giacobbe, S. Giagu, V. Giangiobbe, P. Giannetti, F. Gianotti, B. Gibbard, S. M. Gibson, M. Gignac, M. Gilchriese, T. P. S. Gillam, D. Gillberg, G. Gilles, D. M. Gingrich, N. Giokaris, M. P. Giordani, F. M. Giorgi, F. M. Giorgi, P. F. Giraud, D. Giugni, C. Giuliani, M. Giulini, B. K. Gjelsten, S. Gkaitatzis, I. Gkialas, E. L. Gkougkousis, L. K. Gladilin, C. Glasman, J. Glatzer, P. C. F. Glaysher, A. Glazov, M. Goblirsch-Kolb, J. R. Goddard, J. Godlewski, S. Goldfarb, T. Golling, D. Golubkov, A. Gomes, R. Gonçalo, J. Goncalves Pinto Firmino Da Costa, L. Gonella, S. González de la Hoz, G. Gonzalez Parra, S. Gonzalez-Sevilla, L. Goossens, P. A. Gorbounov, H. A. Gordon, I. Gorelov, B. Gorini, E. Gorini, A. Gorišek, E. Gornicki, A. T. Goshaw, C. Gössling, M. I. Gostkin, M. Gouighri, D. Goujdami, A. G. Goussiou, H. M. X. Grabas, L. Graber, I. Grabowska-Bold, P. Grafström, K-J. Grahn, J. Gramling, E. Gramstad, S. Grancagnolo, V. Grassi, V. Gratchev, H. M. Gray, E. Graziani, Z. D. Greenwood, K. Gregersen, I. M. Gregor, P. Grenier, J. Griffiths, A. A. Grillo, K. Grimm, S. Grinstein, Ph. Gris, Y. V. Grishkevich, J.-F. Grivaz, J. P. Grohs, A. Grohsjean, E. Gross, J. Grosse-Knetter, G. C. Grossi, Z. J. Grout, L. Guan, J. Guenther, F. Guescini, D. Guest, O. Gueta, E. Guido, T. Guillemin, S. Guindon, U. Gul, C. Gumpert, J. Guo, S. Gupta, P. Gutierrez, N. G. Gutierrez Ortiz, C. Gutschow, N. Guttman, C. Guyot, C. Gwenlan, C. B. Gwilliam, A. Haas, C. Haber, H. K. Hadavand, N. Haddad, P. Haefner, S. Hageböck, Z. Hajduk, H. Hakobyan, M. Haleem, J. Haley, D. Hall, G. Halladjian, G. D. Hallewell, K. Hamacher, P. Hamal, K. Hamano, M. Hamer, A. Hamilton, S. Hamilton, G. N. Hamity, P. G. Hamnett, L. Han, K. Hanagaki, K. Hanawa, M. Hance, P. Hanke, R. Hanna, J. B. Hansen, J. D. Hansen, P. H. Hansen, K. Hara, A. S. Hard, T. Harenberg, F. Hariri, S. Harkusha, R. D. Harrington, P. F. Harrison, F. Hartjes, M. Hasegawa, S. Hasegawa, Y. Hasegawa, A. Hasib, S. Hassani, S. Haug, R. Hauser, L. Hauswald, M. Havranek, C. M. Hawkes, R. J. Hawkings, A. D. Hawkins, T. Hayashi, D. Hayden, C. P. Hays, J. M. Hays, H. S. Hayward, S. J. Haywood, S. J. Head, T. Heck, V. Hedberg, L. Heelan, S. Heim, T. Heim, B. Heinemann, L. Heinrich, J. Hejbal, L. Helary, M. Heller, S. Hellman, D. Hellmich, C. Helsens, J. Henderson, R. C. W. Henderson, Y. Heng, C. Hengler, A. Henrichs, A. M. Henriques Correia, S. Henrot-Versille, G. H. Herbert, Y. Hernández Jiménez, R. Herrberg-Schubert, G. Herten, R. Hertenberger, L. Hervas, G. G. Hesketh, N. P. Hessey, R. Hickling, E. Higón-Rodriguez, E. Hill, J. C. Hill, K. H. Hiller, S. J. Hillier, I. Hinchliffe, E. Hines, R. R. Hinman, M. Hirose, D. Hirschbuehl, J. Hobbs, N. Hod, M. C. Hodgkinson, P. Hodgson, A. Hoecker, M. R. Hoeferkamp, F. Hoenig, M. Hohlfeld, T. R. Holmes, T. M. Hong, L. Hooft van Huysduynen, W. H. Hopkins, Y. Horii, A. J. Horton, J-Y. Hostachy, S. Hou, A. Hoummada, J. Howard, J. Howarth, M. Hrabovsky, I. Hristova, J. Hrivnac, T. Hryn’ova, A. Hrynevich, C. Hsu, P. J. Hsu, S.-C. Hsu, D. Hu, Q. Hu, X. Hu, Y. Huang, Z. Hubacek, F. Hubaut, F. Huegging, T. B. Huffman, E. W. Hughes, G. Hughes, M. Huhtinen, T. A. Hülsing, N. Huseynov, J. Huston, J. Huth, G. Iacobucci, G. Iakovidis, I. Ibragimov, L. Iconomidou-Fayard, E. Ideal, Z. Idrissi, P. Iengo, O. Igonkina, T. Iizawa, Y. Ikegami, K. Ikematsu, M. Ikeno, Y. Ilchenko, D. Iliadis, N. Ilic, Y. Inamaru, T. Ince, P. Ioannou, M. Iodice, K. Iordanidou, V. Ippolito, A. Irles Quiles, C. Isaksson, M. Ishino, M. Ishitsuka, R. Ishmukhametov, C. Issever, S. Istin, J. M. Iturbe Ponce, R. Iuppa, J. Ivarsson, W. Iwanski, H. Iwasaki, J. M. Izen, V. Izzo, B. Jackson, M. Jackson, P. Jackson, M. R. Jaekel, V. Jain, K. Jakobs, S. Jakobsen, T. Jakoubek, J. Jakubek, D. O. Jamin, D. K. Jana, E. Jansen, R. W. Jansky, J. Janssen, M. Janus, G. Jarlskog, N. Javadov, T. Javůrek, L. Jeanty, J. Jejelava, G.-Y. Jeng, D. Jennens, P. Jenni, J. Jentzsch, C. Jeske, S. Jézéquel, H. Ji, J. Jia, Y. Jiang, J. Jimenez Pena, S. Jin, A. Jinaru, O. Jinnouchi, M. D. Joergensen, P. Johanssonjiang, K. A. Johns, K. Jon-And, G. Jones, R. W. L. Jones, T. J. Jones, J. Jongmanns, P. M. Jorge, K. D. Joshi, J. Jovicevic, X. Ju, C. A. Jung, P. Jussel, A. Juste Rozas, M. Kaci, A. Kaczmarska, M. Kado, H. Kagan, M. Kagan, S. J. Kahn, E. Kajomovitz, C. W. Kalderon, S. Kama, A. Kamenshchikov, N. Kanaya, M. Kaneda, S. Kaneti, V. A. Kantserov, J. Kanzaki, B. Kaplan, A. Kapliy, D. Kar, K. Karakostas, A. Karamaoun, N. Karastathis, M. J. Kareem, M. Karnevskiy, S. N. Karpov, Z. M. Karpova, K. Karthik, V. Kartvelishvili, A. N. Karyukhin, L. Kashif, R. D. Kass, A. Kastanas, Y. Kataoka, A. Katre, J. Katzy, K. Kawagoe, T. Kawamoto, G. Kawamura, S. Kazama, V. F. Kazanin, M. Y. Kazarinov, R. Keeler, R. Kehoe, M. Keil, J. S. Keller, J. J. Kempster, H. Keoshkerian, O. Kepka, B. P. Kerševan, S. Kersten, R. A. Keyes, F. Khalil-zada, H. Khandanyan, A. Khanov, A. Kharlamov, A. Khodinov, A. Khomich, T. J. Khoo, G. Khoriauli, V. Khovanskiy, E. Khramov, J. Khubua, H. Y. Kim, H. Kim, S. H. Kim, N. Kimura, O. M. Kind, B. T. King, M. King, R. S. B. King, S. B. King, J. Kirk, A. E. Kiryunin, T. Kishimoto, D. Kisielewska, F. Kiss, K. Kiuchi, E. Kladiva, M. Klein, U. Klein, K. Kleinknecht, P. Klimek, A. Klimentov, R. Klingenberg, J. A. Klinger, T. Klioutchnikova, P. F. Klok, E.-E. Kluge, P. Kluit, S. Kluth, E. Kneringer, E. B. F. G. Knoops, A. Knue, D. Kobayashi, T. Kobayashi, M. Kobel, M. Kocian, P. Kodys, T. Koffas, E. Koffeman, L. A. Kogan, S. Kohlmann, Z. Kohout, T. Kohriki, T. Koi, H. Kolanoski, I. Koletsou, A. A. Komar, Y. Komori, T. Kondo, N. Kondrashova, K. Köneke, A. C. König, S. König, T. Kono, R. Konoplich, N. Konstantinidis, R. Kopeliansky, S. Koperny, L. Köpke, A. K. Kopp, K. Korcyl, K. Kordas, A. Korn, A. A. Korol, I. Korolkov, E. V. Korolkova, O. Kortner, S. Kortner, T. Kosek, V. V. Kostyukhin, V. M. Kotov, A. Kotwal, A. Kourkoumeli-Charalampidi, C. Kourkoumelis, V. Kouskoura, A. Koutsman, R. Kowalewski, T. Z. Kowalski, W. Kozanecki, A. S. Kozhin, V. A. Kramarenko, G. Kramberger, D. Krasnopevtsev, M. W. Krasny, A. Krasznahorkay, J. K. Kraus, A. Kravchenko, S. Kreiss, M. Kretz, J. Kretzschmar, K. Kreutzfeldt, P. Krieger, K. Krizka, K. Kroeninger, H. Kroha, J. Kroll, J. Kroseberg, J. Krstic, U. Kruchonak, H. Krüger, N. Krumnack, Z. V. Krumshteyn, A. Kruse, M. C. Kruse, M. Kruskal, T. Kubota, H. Kucuk, S. Kuday, S. Kuehn, A. Kugel, F. Kuger, A. Kuhl, T. Kuhl, V. Kukhtin, Y. Kulchitsky, S. Kuleshov, M. Kuna, T. Kunigo, A. Kupco, H. Kurashige, Y. A. Kurochkin, R. Kurumida, V. Kus, E. S. Kuwertz, M. Kuze, J. Kvita, T. Kwan, D. Kyriazopoulos, A. La Rosa, J. L. La Rosa Navarro, L. La Rotonda, C. Lacasta, F. Lacava, J. Lacey, H. Lacker, D. Lacour, V. R. Lacuesta, E. Ladygin, R. Lafaye, B. Laforge, T. Lagouri, S. Lai, L. Lambourne, S. Lammers, C. L. Lampen, W. Lampl, E. Lançon, U. Landgraf, M. P. J. Landon, V. S. Lang, A. J. Lankford, F. Lanni, K. Lantzsch, S. Laplace, C. Lapoire, J. F. Laporte, T. Lari, F. Lasagni Manghi, M. Lassnig, P. Laurelli, W. Lavrijsen, A. T. Law, P. Laycock, O. Le Dortz, E. Le Guirriec, E. Le Menedeu, T. LeCompte, F. Ledroit-Guillon, C. A. Lee, S. C. Lee, L. Lee, G. Lefebvre, M. Lefebvre, F. Legger, C. Leggett, A. Lehan, G. Lehmann Miotto, X. Lei, W. A. Leight, A. Leisos, A. G. Leister, M. A. L. Leite, R. Leitner, D. Lellouch, B. Lemmer, K. J. C. Leney, T. Lenz, G. Lenzen, B. Lenzi, R. Leone, S. Leone, C. Leonidopoulos, S. Leontsinis, C. Leroy, C. G. Lester, M. Levchenko, J. Levêque, D. Levin, L. J. Levinson, M. Levy, A. Lewis, A. M. Leyko, M. Leyton, B. Li, B. Li, H. Li, H. L. Li, L. Li, L. Li, S. Li, Y. Li, Z. Liang, H. Liao, B. Liberti, P. Lichard, K. Lie, J. Liebal, W. Liebig, C. Limbach, A. Limosani, S. C. Lin, T. H. Lin, F. Linde, B. E. Lindquist, J. T. Linnemann, E. Lipeles, A. Lipniacka, M. Lisovyi, T. M. Liss, D. Lissauer, A. Lister, A. M. Litke, B. Liu, D. Liu, J. Liu, J. B. Liu, K. Liu, L. Liu, M. Liu, M. Liu, Y. Liu, M. Livan, A. Lleres, J. Llorente Merino, S. L. Lloyd, F. Lo Sterzo, E. Lobodzinska, P. Loch, W. S. Lockman, F. K. Loebinger, A. E. Loevschall-Jensen, A. Loginov, T. Lohse, K. Lohwasser, M. Lokajicek, B. A. Long, J. D. Long, R. E. Long, K. A. Looper, L. Lopes, D. Lopez Mateos, B. Lopez Paredes, I. Lopez Paz, J. Lorenz, N. Lorenzo Martinez, M. Losada, P. Loscutoff, P. J. Lösel, X. Lou, A. Lounis, J. Love, P. A. Love, F. Lu, N. Lu, H. J. Lubatti, C. Luci, A. Lucotte, F. Luehring, W. Lukas, L. Luminari, O. Lundberg, B. Lund-Jensen, M. Lungwitz, D. Lynn, R. Lysak, E. Lytken, H. Ma, L. L. Ma, G. Maccarrone, A. Macchiolo, J. Machado Miguens, D. Macina, D. Madaffari, R. Madar, H. J. Maddocks, W. F. Mader, A. Madsen, T. Maeno, A. Maevskiy, E. Magradze, K. Mahboubi, J. Mahlstedt, S. Mahmoud, C. Maiani, C. Maidantchik, A. A. Maier, A. Maio, S. Majewski, Y. Makida, N. Makovec, B. Malaescu, Pa. Malecki, V. P. Maleev, F. Malek, U. Mallik, D. Malon, C. Malone, S. Maltezos, V. M. Malyshev, S. Malyukov, J. Mamuzic, B. Mandelli, L. Mandelli, I. Mandić, R. Mandrysch, J. Maneira, A. Manfredini, L. Manhaes de Andrade Filho, J. Majarres Ramos, A. Mann, P. M. Manning, A. Manousakis-Katsikakis, B. Mansoulie, R. Mantifel, M. Mantoani, L. Mapelli, L. March, G. Marchiori, M. Marcisovsky, C. P. Marino, M. Marjanovic, F. Marroquim, S. P. Marsden, Z. Marshall, L. F. Marti, S. Marti-Garcia, B. Martin, T. A. Martin, V. J. Martin, B. Martin dit Latour, H. Martinez, M. Martinez, S. Martin-Haugh, A. C. Martyniuk, M. Marx, F. Marzano, A. Marzin, L. Masetti, T. Mashimo, R. Mashinistov, J. Masik, A. L. Maslennikov, I. Massa, L. Massa, N. Massol, P. Mastrandrea, A. Mastroberardino, T. Masubuchi, P. Mättig, J. Mattmann, J. Maurer, S. J. Maxfield, D. A. Maximov, R. Mazini, S. M. Mazza, L. Mazzaferro, G. Mc Goldrick, S. P. Mc Kee, A. McCarn, R. L. McCarthy, T. G. McCarthy, N. A. McCubbin, K. W. McFarlane, J. A. Mcfayden, G. Mchedlidze, S. J. McMahon, R. A. McPherson, J. Mechnich, M. Medinnis, S. Meehan, S. Mehlhase, A. Mehta, K. Meier, C. Meineck, B. Meirose, C. Melachrinos, B. R. Mellado Garcia, F. Meloni, A. Mengarelli, S. Menke, E. Meoni, K. M. Mercurio, S. Mergelmeyer, N. Meric, P. Mermod, L. Merola, C. Meroni, F. S. Merritt, H. Merritt, A. Messina, J. Metcalfe, A. S. Mete, C. Meyer, C. Meyer, J-P. Meyer, J. Meyer, R. P. Middleton, S. Migas, S. Miglioranzi, L. Mijović, G. Mikenberg, M. Mikestikova, M. Mikuž, A. Milic, D. W. Miller, C. Mills, A. Milov, D. A. Milstead, A. A. Minaenko, Y. Minami, I. A. Minashvili, A. I. Mincer, B. Mindur, M. Mineev, Y. Ming, L. M. Mir, G. Mirabelli, T. Mitani, J. Mitrevski, V. A. Mitsou, A. Miucci, P. S. Miyagawa, J. U. Mjörnmark, T. Moa, K. Mochizuki, S. Mohapatra, W. Mohr, S. Molander, R. Moles-Valls, K. Mönig, C. Monini, J. Monk, E. Monnier, J. Montejo Berlingen, F. Monticelli, S. Monzani, R. W. Moore, N. Morange, D. Moreno, M. Moreno Llácer, P. Morettini, M. Morgenstern, M. Morii, V. Morisbak, S. Moritz, A. K. Morley, G. Mornacchi, J. D. Morris, A. Morton, L. Morvaj, H. G. Moser, M. Mosidze, J. Moss, K. Motohashi, R. Mount, E. Mountricha, S. V. Mouraviev, E. J. W. Moyse, S. Muanza, R. D. Mudd, F. Mueller, J. Mueller, K. Mueller, R. S. P. Mueller, T. Mueller, D. Muenstermann, P. Mullen, Y. Munwes, J. A. Murillo Quijada, W. J. Murray, H. Musheghyan, E. Musto, A. G. Myagkov, M. Myska, O. Nackenhorst, J. Nadal, K. Nagai, R. Nagai, Y. Nagai, K. Nagano, A. Nagarkar, Y. Nagasaka, K. Nagata, M. Nagel, E. Nagy, A. M. Nairz, Y. Nakahama, K. Nakamura, T. Nakamura, I. Nakano, H. Namasivayam, G. Nanava, R. F. Naranjo Garcia, R. Narayan, T. Nattermann, T. Naumann, G. Navarro, R. Nayyar, H. A. Neal, P. Yu. Nechaeva, T. J. Neep, P. D. Nef, A. Negri, M. Negrini, S. Nektarijevic, C. Nellist, A. Nelson, S. Nemecek, P. Nemethy, A. A. Nepomuceno, M. Nessi, M. S. Neubauer, M. Neumann, R. M. Neves, P. Nevski, P. R. Newman, D. H. Nguyen, R. B. Nickerson, R. Nicolaidou, B. Nicquevert, J. Nielsen, N. Nikiforou, A. Nikiforov, V. Nikolaenko, I. Nikolic-Audit, K. Nikolopoulos, P. Nilsson, Y. Ninomiya, A. Nisati, R. Nisius, T. Nobe, M. Nomachi, I. Nomidis, S. Norberg, M. Nordberg, O. Novgorodova, S. Nowak, M. Nozaki, L. Nozka, K. Ntekas, G. Nunes Hanninger, T. Nunnemann, E. Nurse, F. Nuti, B. J. O’Brien, F. O’grady, D. C. O’Neil, V. O’Shea, F. G. Oakham, H. Oberlack, T. Obermann, J. Ocariz, A. Ochi, I. Ochoa, S. Oda, S. Odaka, H. Ogren, A. Oh, S. H. Oh, C. C. Ohm, H. Ohman, H. Oide, W. Okamura, H. Okawa, Y. Okumura, T. Okuyama, A. Olariu, A. G. Olchevski, S. A. Olivares Pino, D. Oliveira Damazio, E. Oliver Garcia, A. Olszewski, J. Olszowska, A. Onofre, P. U. E. Onyisi, C. J. Oram, M. J. Oreglia, Y. Oren, D. Orestano, N. Orlando, C. Oropeza Barrera, R. S. Orr, B. Osculati, R. Ospanov, G. Otero y Garzon, H. Otono, M. Ouchrif, E. A. Ouellette, F. Ould-Saada, A. Ouraou, K. P. Oussoren, Q. Ouyang, A. Ovcharova, M. Owen, R. E. Owen, V. E. Ozcan, N. Ozturk, K. Pachal, A. Pacheco Pages, C. Padilla Aranda, M. Pagáčová, S. Pagan Griso, E. Paganis, C. Pahl, F. Paige, P. Pais, K. Pajchel, G. Palacino, S. Palestini, M. Palka, D. Pallin, A. Palma, Y. B. Pan, E. Panagiotopoulou, C. E. Pandini, J. G. Panduro Vazquez, P. Pani, N. Panikashvili, S. Panitkin, L. Paolozzi, Th. D. Papadopoulou, K. Papageorgiou, A. Paramonov, D. Paredes Hernandez, M. A. Parker, K. A. Parker, F. Parodi, J. A. Parsons, U. Parzefall, E. Pasqualucci, S. Passaggio, F. Pastore, Fr. Pastore, G. Pásztor, S. Pataraia, N. D. Patel, J. R. Pater, T. Pauly, J. Pearce, L. E. Pedersen, M. Pedersen, S. Pedraza Lopez, R. Pedro, S. V. Peleganchuk, S. V. Peleganchuk, D. Pelikan, H. Peng, B. Penning, J. Penwell, D. V. Perepelitsa, E. Perez Codina, M. T. Pérez García-Estañ, L. Perini, H. Pernegger, S. Perrella, R. Peschke, V. D. Peshekhonov, K. Peters, R. F. Y. Peters, B. A. Petersen, T. C. Petersen, E. Petit, A. Petridis, C. Petridou, E. Petrolo, F. Petrucci, N. E. Pettersson, R. Pezoa, P. W. Phillips, G. Piacquadio, E. Pianori, A. Picazio, E. Piccaro, M. Piccinini, M. A. Pickering, R. Piegaia, D. T. Pignotti, J. E. Pilcher, A. D. Pilkington, J. Pina, M. Pinamonti, J. L. Pinfold, A. Pingel, B. Pinto, S. Pires, M. Pitt, C. Pizio, L. Plazak, M.-A. Pleier, V. Pleskot, E. Plotnikova, P. Plucinski, D. Pluth, S. Poddar, R. Poettgen, L. Poggioli, D. Pohl, G. Polesello, A. Policicchio, R. Polifka, A. Polini, C. S. Pollard, V. Polychronakos, K. Pommès, L. Pontecorvo, B. G. Pope, G. A. Popeneciu, D. S. Popovic, A. Poppleton, S. Pospisil, K. Potamianos, I. N. Potrap, C. J. Potter, C. T. Potter, G. Poulard, J. Poveda, V. Pozdnyakov, P. Pralavorio, A. Pranko, S. Prasad, S. Prell, D. Price, J. Price, L. E. Price, M. Primavera, S. Prince, M. Proissl, K. Prokofiev, F. Prokoshin, E. Protopapadaki, S. Protopopescu, J. Proudfoot, M. Przybycien, E. Ptacek, D. Puddu, E. Pueschel, D. Puldon, M. Purohit, P. Puzo, J. Qian, G. Qin, Y. Qin, A. Quadt, D. R. Quarrie, W. B. Quayle, M. Queitsch-Maitland, D. Quilty, A. Qureshi, V. Radeka, V. Radescu, S. K. Radhakrishnan, P. Radloff, P. Rados, F. Ragusa, G. Rahal, S. Rajagopalan, M. Rammensee, C. Rangel-Smith, F. Rauscher, S. Rave, T. C. Rave, T. Ravenscroft, M. Raymond, A. L. Read, N. P. Readioff, D. M. Rebuzzi, A. Redelbach, G. Redlinger, R. Reece, K. Reeves, L. Rehnisch, H. Reisin, M. Relich, C. Rembser, H. Ren, A. Renaud, M. Rescigno, S. Resconi, O. L. Rezanova, P. Reznicek, R. Rezvani, R. Richter, E. Richter-Was, M. Ridel, P. Rieck, C. J. Riegel, J. Rieger, M. Rijssenbeek, A. Rimoldi, L. Rinaldi, E. Ritsch, I. Riu, F. Rizatdinova, E. Rizvi, S. H. Robertson, A. Robichaud-Veronneau, D. Robinson, J. E. M. Robinson, A. Robson, C. Roda, L. Rodrigues, S. Roe, O. Røhne, S. Rolli, A. Romaniouk, M. Romano, S. M. Romano Saez, E. Romero Adam, N. Rompotis, M. Ronzani, L. Roos, E. Ros, S. Rosati, K. Rosbach, P. Rose, P. L. Rosendahl, O. Rosenthal, V. Rossetti, E. Rossi, L. P. Rossi, R. Rosten, M. Rotaru, I. Roth, J. Rothberg, D. Rousseau, C. R. Royon, A. Rozanov, Y. Rozen, X. Ruan, F. Rubbo, I. Rubinskiy, V. I. Rud, C. Rudolph, M. S. Rudolph, F. Rühr, A. Ruiz-Martinez, Z. Rurikova, N. A. Rusakovich, A. Ruschke, H. L. Russell, J. P. Rutherfoord, N. Ruthmann, Y. F. Ryabov, M. Rybar, G. Rybkin, N. C. Ryder, A. F. Saavedra, G. Sabato, S. Sacerdoti, A. Saddique, H. F-W. Sadrozinski, R. Sadykov, F. Safai Tehrani, M. Saimpert, H. Sakamoto, Y. Sakurai, G. Salamanna, A. Salamon, M. Saleem, D. Salek, P. H. Sales De Bruin, D. Salihagic, A. Salnikov, J. Salt, D. Salvatore, F. Salvatore, A. Salvucci, A. Salzburger, D. Sampsonidis, A. Sanchez, J. Sánchez, V. Sanchez Martinez, H. Sandaker, R. L. Sandbach, H. G. Sander, M. P. Sanders, M. Sandhoff, C. Sandoval, R. Sandstroem, D. P. C. Sankey, A. Sansoni, C. Santoni, R. Santonico, H. Santos, I. Santoyo Castillo, K. Sapp, A. Sapronov, J. G. Saraiva, B. Sarrazin, O. Sasaki, Y. Sasaki, K. Sato, G. Sauvage, E. Sauvan, G. Savage, P. Savard, C. Sawyer, L. Sawyer, D. H. Saxon, J. Saxon, C. Sbarra, A. Sbrizzi, T. Scanlon, D. A. Scannicchio, M. Scarcella, V. Scarfone, J. Schaarschmidt, P. Schacht, D. Schaefer, R. Schaefer, J. Schaeffer, S. Schaepe, S. Schaetzel, U. Schäfer, A. C. Schaffer, D. Schaile, R. D. Schamberger, V. Scharf, V. A. Schegelsky, D. Scheirich, M. Schernau, C. Schiavi, C. Schillo, M. Schioppa, S. Schlenker, E. Schmidt, K. Schmieden, C. Schmitt, S. Schmitt, B. Schneider, Y. J. Schnellbach, U. Schnoor, L. Schoeffel, A. Schoening, B. D. Schoenrock, A. L. S. Schorlemmer, M. Schott, D. Schouten, J. Schovancova, S. Schramm, M. Schreyer, C. Schroeder, N. Schuh, M. J. Schultens, H.-C. Schultz-Coulon, H. Schulz, M. Schumacher, B. A. Schumm, Ph. Schune, C. Schwanenberger, A. Schwartzman, T. A. Schwarz, Ph. Schwegler, Ph. Schwemling, R. Schwienhorst, J. Schwindling, T. Schwindt, M. Schwoerer, F. G. Sciacca, E. Scifo, G. Sciolla, F. Scuri, F. Scutti, J. Searcy, G. Sedov, E. Sedykh, P. Seema, S. C. Seidel, A. Seiden, F. Seifert, J. M. Seixas, G. Sekhniaidze, S. J. Sekula, K. E. Selbach, D. M. Seliverstov, N. Semprini-Cesari, C. Serfon, L. Serin, L. Serkin, T. Serre, R. Seuster, H. Severini, T. Sfiligoj, F. Sforza, A. Sfyrla, E. Shabalina, M. Shamim, L. Y. Shan, R. Shang, J. T. Shank, M. Shapiro, P. B. Shatalov, K. Shaw, A. Shcherbakova, C. Y. Shehu, P. Sherwood, L. Shi, S. Shimizu, C. O. Shimmin, M. Shimojima, M. Shiyakova, A. Shmeleva, D. Shoaleh Saadi, M. J. Shochet, S. Shojaii, S. Shrestha, E. Shulga, M. A. Shupe, S. Shushkevich, P. Sicho, O. Sidiropoulou, D. Sidorov, A. Sidoti, F. Siegert, Dj. Sijacki, J. Silva, Y. Silver, D. Silverstein, S. B. Silverstein, V. Simak, O. Simard, Lj. Simic, S. Simion, E. Simioni, B. Simmons, D. Simon, R. Simoniello, P. Sinervo, N. B. Sinev, G. Siragusa, A. Sircar, A. N. Sisakyan, S. Yu. Sivoklokov, J. Sjölin, T. B. Sjursen, H. P. Skottowe, P. Skubic, M. Slater, T. Slavicek, M. Slawinska, K. Sliwa, V. Smakhtin, B. H. Smart, L. Smestad, S. Yu. Smirnov, Y. Smirnov, L. N. Smirnova, O. Smirnova, K. M. Smith, M. N. K. Smith, M. Smizanska, K. Smolek, A. A. Snesarev, G. Snidero, S. Snyder, R. Sobie, F. Socher, A. Soffer, D. A. Soh, C. A. Solans, M. Solar, J. Solc, E. Yu. Soldatov, U. Soldevila, A. A. Solodkov, A. Soloshenko, O. V. Solovyanov, V. Solovyev, P. Sommer, H. Y. Song, N. Soni, A. Sood, A. Sopczak, B. Sopko, V. Sopko, V. Sorin, D. Sosa, M. Sosebee, C. L. Sotiropoulou, R. Soualah, P. Soueid, A. M. Soukharev, D. South, S. Spagnolo, F. Spanò, W. R. Spearman, F. Spettel, R. Spighi, G. Spigo, L. A. Spiller, M. Spousta, T. Spreitzer, R. D. St. Denis, S. Staerz, J. Stahlman, R. Stamen, S. Stamm, E. Stanecka, C. Stanescu, M. Stanescu-Bellu, M. M. Stanitzki, S. Stapnes, E. A. Starchenko, J. Stark, P. Staroba, P. Starovoitov, R. Staszewski, P. Stavina, P. Steinberg, B. Stelzer, H. J. Stelzer, O. Stelzer-Chilton, H. Stenzel, S. Stern, G. A. Stewart, J. A. Stillings, M. C. Stockton, M. Stoebe, G. Stoicea, P. Stolte, S. Stonjek, A. R. Stradling, A. Straessner, M. E. Stramaglia, J. Strandberg, S. Strandberg, A. Strandlie, E. Strauss, M. Strauss, P. Strizenec, R. Ströhmer, D. M. Strom, R. Stroynowski, A. Strubig, S. A. Stucci, B. Stugu, N. A. Styles, D. Su, J. Su, R. Subramaniam, A. Succurro, Y. Sugaya, C. Suhr, M. Suk, V. V. Sulin, S. Sultansoy, T. Sumida, S. Sun, X. Sun, J. E. Sundermann, K. Suruliz, G. Susinno, M. R. Sutton, Y. Suzuki, M. Svatos, S. Swedish, M. Swiatlowski, I. Sykora, T. Sykora, D. Ta, C. Taccini, K. Tackmann, J. Taenzer, A. Taffard, R. Tafirout, N. Taiblum, H. Takai, R. Takashima, H. Takeda, T. Takeshita, Y. Takubo, M. Talby, A. A. Talyshev, J. Y. C. Tam, K. G. Tan, J. Tanaka, R. Tanaka, S. Tanaka, S. Tanaka, A. J. Tanasijczuk, B. B. Tannenwald, N. Tannoury, S. Tapprogge, S. Tarem, F. Tarrade, G. F. Tartarelli, P. Tas, M. Tasevsky, T. Tashiro, E. Tassi, A. Tavares Delgado, Y. Tayalati, F. E. Taylor, G. N. Taylor, W. Taylor, F. A. Teischinger, M. Teixeira Dias Castanheira, P. Teixeira-Dias, K. K. Temming, H. Ten Kate, P. K. Teng, J. J. Teoh, F. Tepel, S. Terada, K. Terashi, J. Terron, S. Terzo, M. Testa, R. J. Teuscher, J. Therhaag, T. Theveneaux-Pelzer, J. P. Thomas, J. Thomas-Wilsker, E. N. Thompson, P. D. Thompson, R. J. Thompson, A. S. Thompson, L. A. Thomsen, E. Thomson, M. Thomson, W. M. Thong, R. P. Thun, F. Tian, M. J. Tibbetts, R. E. Ticse Torres, V. O. Tikhomirov, Yu. A. Tikhonov, S. Timoshenko, E. Tiouchichine, P. Tipton, S. Tisserant, T. Todorov, S. Todorova-Nova, J. Tojo, S. Tokár, K. Tokushuku, K. Tollefson, E. Tolley, L. Tomlinson, M. Tomoto, L. Tompkins, K. Toms, N. D. Topilin, E. Torrence, H. Torres, E. Torró Pastor, J. Toth, F. Touchard, D. R. Tovey, H. L. Tran, T. Trefzger, L. Tremblet, A. Tricoli, I. M. Trigger, S. Trincaz-Duvoid, M. F. Tripiana, W. Trischuk, B. Trocmé, C. Troncon, M. Trottier-McDonald, M. Trovatelli, P. True, M. Trzebinski, A. Trzupek, C. Tsarouchas, J. C-L. Tseng, P. V. Tsiareshka, D. Tsionou, G. Tsipolitis, N. Tsirintanis, S. Tsiskaridze, V. Tsiskaridze, E. G. Tskhadadze, I. I. Tsukerman, V. Tsulaia, S. Tsuno, D. Tsybychev, A. Tudorache, V. Tudorache, A. N. Tuna, S. A. Tupputi, S. Turchikhin, D. Turecek, I. Turk Cakir, R. Turra, A. J. Turvey, P. M. Tuts, A. Tykhonov, M. Tylmad, M. Tyndel, I. Ueda, R. Ueno, M. Ughetto, M. Ugland, M. Uhlenbrock, F. Ukegawa, G. Unal, A. Undrus, G. Unel, F. C. Ungaro, Y. Unno, C. Unverdorben, J. Urban, P. Urquijo, P. Urrejola, G. Usai, A. Usanova, L. Vacavant, V. Vacek, B. Vachon, N. Valencic, S. Valentinetti, A. Valero, L. Valery, S. Valkar, E. Valladolid Gallego, S. Vallecorsa, J. A. Valls Ferrer, W. Van Den Wollenberg, P. C. Van Der Deijl, R. van der Geer, H. van der Graaf, R. Van Der Leeuw, N. van Eldik, P. van Gemmeren, J. Van Nieuwkoop, I. van Vulpen, M. C. van Woerden, M. Vanadia, W. Vandelli, R. Vanguri, A. Vaniachine, F. Vannucci, G. Vardanyan, R. Vari, E. W. Varnes, T. Varol, D. Varouchas, A. Vartapetian, K. E. Varvell, F. Vazeille, T. Vazquez Schroeder, J. Veatch, F. Veloso, T. Velz, S. Veneziano, A. Ventura, D. Ventura, M. Venturi, N. Venturi, A. Venturini, V. Vercesi, M. Verducci, W. Verkerke, J. C. Vermeulen, A. Vest, M. C. Vetterli, O. Viazlo, I. Vichou, T. Vickey, O. E. Vickey Boeriu, G. H. A. Viehhauser, S. Viel, R. Vigne, M. Villa, M. Villaplana Perez, E. Vilucchi, M. G. Vincter, V. B. Vinogradov, J. Virzi, I. Vivarelli, F. Vives Vaque, S. Vlachos, D. Vladoiu, M. Vlasak, M. Vogel, P. Vokac, G. Volpi, M. Volpi, H. von der Schmitt, H. von Radziewski, E. von Toerne, V. Vorobel, K. Vorobev, M. Vos, R. Voss, J. H. Vossebeld, N. Vranjes, M. Vranjes Milosavljevic, V. Vrba, M. Vreeswijk, R. Vuillermet, I. Vukotic, Z. Vykydal, P. Wagner, W. Wagner, H. Wahlberg, S. Wahrmund, J. Wakabayashi, J. Walder, R. Walker, W. Walkowiak, C. Wang, F. Wang, H. Wang, H. Wang, J. Wang, J. Wang, K. Wang, R. Wang, S. M. Wang, T. Wang, X. Wang, C. Wanotayaroj, A. Warburton, C. P. Ward, D. R. Wardrope, M. Warsinsky, A. Washbrook, C. Wasicki, P. M. Watkins, A. T. Watson, I. J. Watson, M. F. Watson, G. Watts, S. Watts, B. M. Waugh, S. Webb, M. S. Weber, S. W. Weber, J. S. Webster, A. R. Weidberg, B. Weinert, J. Weingarten, C. Weiser, H. Weits, P. S. Wells, T. Wenaus, D. Wendland, T. Wengler, S. Wenig, N. Wermes, M. Werner, P. Werner, M. Wessels, J. Wetter, K. Whalen, A. M. Wharton, A. White, M. J. White, R. White, S. White, D. Whiteson, D. Wicke, F. J. Wickens, W. Wiedenmann, M. Wielers, P. Wienemann, C. Wiglesworth, L. A. M. Wiik-Fuchs, A. Wildauer, H. G. Wilkens, H. H. Williams, S. Williams, C. Willis, S. Willocq, A. Wilson, J. A. Wilson, I. Wingerter-Seez, F. Winklmeier, B. T. Winter, M. Wittgen, J. Wittkowski, S. J. Wollstadt, M. W. Wolter, H. Wolters, B. K. Wosiek, J. Wotschack, M. J. Woudstra, K. W. Wozniak, M. Wu, S. L. Wu, X. Wu, Y. Wu, T. R. Wyatt, B. M. Wynne, S. Xella, D. Xu, L. Xu, B. Yabsley, S. Yacoob, R. Yakabe, M. Yamada, Y. Yamaguchi, A. Yamamoto, S. Yamamoto, T. Yamanaka, K. Yamauchi, Y. Yamazaki, Z. Yan, H. Yang, H. Yang, Y. Yang, S. Yanush, L. Yao, W-M. Yao, Y. Yasu, E. Yatsenko, K. H. Yau Wong, J. Ye, S. Ye, I. Yeletskikh, A. L. Yen, E. Yildirim, K. Yorita, R. Yoshida, K. Yoshihara, C. Young, C. J. S. Young, S. Youssef, D. R. Yu, J. Yu, J. M. Yu, J. Yu, L. Yuan, A. Yurkewicz, I. Yusuff, B. Zabinski, R. Zaidan, A. M. Zaitsev, A. Zaman, S. Zambito, L. Zanello, D. Zanzi, C. Zeitnitz, M. Zeman, A. Zemla, K. Zengel, O. Zenin, T. Ženiš, D. Zerwas, D. Zhang, F. Zhang, J. Zhang, L. Zhang, R. Zhang, X. Zhang, Z. Zhang, X. Zhao, Y. Zhao, Z. Zhao, A. Zhemchugov, J. Zhong, B. Zhou, C. Zhou, L. Zhou, L. Zhou, N. Zhou, C. G. Zhu, H. Zhu, J. Zhu, Y. Zhu, X. Zhuang, K. Zhukov, A. Zibell, D. Zieminska, N. I. Zimine, C. Zimmermann, R. Zimmermann, S. Zimmermann, Z. Zinonos, M. Zinser, M. Ziolkowski, L. Živković, G. Zobernig, A. Zoccoli, M. zur Nedden, G. Zurzolo, L. Zwalinski, ATLAS Collaboration

**Affiliations:** Department of Physics, University of Adelaide, Adelaide, Australia; Physics Department, SUNY Albany, Albany, NY USA; Department of Physics, University of Alberta, Edmonton, AB Canada; Department of Physics, Ankara University, Ankara, Turkey, Ankara, Turkey; LAPP, CNRS/IN2P3 and Université de Savoie, Annecy-le-Vieux, France; High Energy Physics Division, Argonne National Laboratory, Argonne, IL USA; Department of Physics, University of Arizona, Tucson, AZ USA; Department of Physics, The University of Texas at Arlington, Arlington, TX USA; Physics Department, University of Athens, Athens, Greece; Physics Department, National Technical University of Athens, Zografou, Greece; Institute of Physics, Azerbaijan Academy of Sciences, Baku, Azerbaijan; Institut de Física d’Altes Energies and Departament de Física de la Universitat Autònoma de Barcelona, Barcelona, Spain; Institute of Physics, University of Belgrade, Belgrade, Serbia; Department for Physics and Technology, University of Bergen, Bergen, Norway; Physics Division, Lawrence Berkeley National Laboratory and University of California, Berkeley, CA USA; Department of Physics, Humboldt University, Berlin, Germany; Albert Einstein Center for Fundamental Physics and Laboratory for High Energy Physics, University of Bern, Bern, Switzerland; School of Physics and Astronomy, University of Birmingham, Birmingham, UK; Department of Physics, Bogazici University, Istanbul, Turkey; INFN Sezione di Bologna, Bologna, Italy; Physikalisches Institut, University of Bonn, Bonn, Germany; Department of Physics, Boston University, Boston, MA USA; Department of Physics, Brandeis University, Waltham, MA USA; Universidade Federal do Rio De Janeiro COPPE/EE/IF, Rio de Janeiro, Brazil; Physics Department, Brookhaven National Laboratory, Upton, NY USA; National Institute of Physics and Nuclear Engineering, Bucharest, Romania; Departamento de Física, Universidad de Buenos Aires, Buenos Aires, Argentina; Cavendish Laboratory, University of Cambridge, Cambridge, UK; Department of Physics, Carleton University, Ottawa, ON Canada; CERN, Geneva, Switzerland; Enrico Fermi Institute, University of Chicago, Chicago, IL USA; Departamento de Física, Pontificia Universidad Católica de Chile, Santiago, Chile; Institute of High Energy Physics, Chinese Academy of Sciences, Beijing, China; Laboratoire de Physique Corpusculaire, Clermont Université and Université Blaise Pascal and CNRS/IN2P3, Clermont-Ferrand, France; Nevis Laboratory, Columbia University, Irvington, NY USA; Niels Bohr Institute, University of Copenhagen, Copenhagen, Denmark; Laboratori Nazionali di Frascati, INFN Gruppo Collegato di Cosenza, Frascati, Italy; Faculty of Physics and Applied Computer Science, AGH University of Science and Technology, Kraków, Poland; Institute of Nuclear Physics, Polish Academy of Sciences, Kraków, Poland; Physics Department, Southern Methodist University, Dallas, TX USA; Physics Department, University of Texas at Dallas, Richardson, TX USA; DESY, Hamburg and Zeuthen, Germany; Institut für Experimentelle Physik IV, Technische Universität Dortmund, Dortmund, Germany; Institut für Kern- und Teilchenphysik, Technische Universität Dresden, Dresden, Germany; Department of Physics, Duke University, Durham, NC USA; SUPA, School of Physics and Astronomy, University of Edinburgh, Edinburgh, UK; INFN Laboratori Nazionali di Frascati, Frascati, Italy; Fakultät für Mathematik und Physik, Albert-Ludwigs-Universität, Freiburg, Germany; Section de Physique, Université de Genève, Geneva, Switzerland; INFN Sezione di Genova, Genoa, Italy; E. Andronikashvili Institute of Physics, Iv. Javakhishvili Tbilisi State University, Tbilisi, Georgia; II Physikalisches Institut, Justus-Liebig-Universität Giessen, Giessen, Germany; SUPA, School of Physics and Astronomy, University of Glasgow, Glasgow, UK; II Physikalisches Institut, Georg-August-Universität, Göttingen, Germany; Laboratoire de Physique Subatomique et de Cosmologie, Université Grenoble-Alpes, CNRS/IN2P3, Grenoble, France; Department of Physics, Hampton University, Hampton, VA USA; Laboratory for Particle Physics and Cosmology, Harvard University, Cambridge, MA USA; Kirchhoff-Institut für Physik , Ruprecht-Karls-Universität Heidelberg, Heidelberg, Germany; Faculty of Applied Information Science, Hiroshima Institute of Technology, Hiroshima, Japan; Department of Physics, The Chinese University of Hong Kong, Shatin, New Territories, Hong Kong, China; Department of Physics, Indiana University, Bloomington, IN USA; Institut für Astro- und Teilchenphysik, Leopold-Franzens-Universität, Innsbruck, Austria; University of Iowa, Iowa City, IA USA; Department of Physics and Astronomy, Iowa State University, Ames, IA USA; Joint Institute for Nuclear Research, JINR Dubna, Dubna, Russia; KEK, High Energy Accelerator Research Organization, Tsukuba, Japan; Graduate School of Science, Kobe University, Kobe, Japan; Faculty of Science, Kyoto University, Kyoto, Japan; Kyoto University of Education, Kyoto, Japan; Department of Physics, Kyushu University, Fukuoka, Japan; Instituto de Física La Plata, Universidad Nacional de La Plata and CONICET, La Plata, Argentina; Physics Department, Lancaster University, Lancaster, UK; INFN Sezione di Lecce, Lecce, Italy; Oliver Lodge Laboratory, University of Liverpool, Liverpool, UK; Department of Physics, Jožef Stefan Institute and University of Ljubljana, Ljubljana, Slovenia; School of Physics and Astronomy, Queen Mary University of London, London, UK; Department of Physics, Royal Holloway University of London, Surrey, UK; Department of Physics and Astronomy, University College London, London, UK; Louisiana Tech University, Ruston, LA USA; Laboratoire de Physique Nucléaire et de Hautes Energies, UPMC and Université Paris-Diderot and CNRS/IN2P3, Paris, France; Fysiska institutionen, Lunds universitet, Lund, Sweden; Departamento de Fisica Teorica C-15, Universidad Autonoma de Madrid, Madrid, Spain; Institut für Physik, Universität Mainz, Mainz, Germany; School of Physics and Astronomy, University of Manchester, Manchester, UK; CPPM, Aix-Marseille Université and CNRS/IN2P3, Marseille, France; Department of Physics, University of Massachusetts, Amherst, MA USA; Department of Physics, McGill University, Montreal, QC Canada; School of Physics, University of Melbourne, Melbourne, VIC Australia; Department of Physics, The University of Michigan, Ann Arbor, MI USA; Department of Physics and Astronomy, Michigan State University, East Lansing, MI USA; INFN Sezione di Milano, Milan, Italy; B.I. Stepanov Institute of Physics, National Academy of Sciences of Belarus, Minsk, Republic of Belarus; National Scientific and Educational Centre for Particle and High Energy Physics, Minsk, Republic of Belarus; Department of Physics, Massachusetts Institute of Technology, Cambridge, MA USA; Group of Particle Physics, University of Montreal, Montreal, QC Canada; P.N. Lebedev Institute of Physics, Academy of Sciences, Moscow, Russia; Institute for Theoretical and Experimental Physics (ITEP), Moscow, Russia; National Research Nuclear University MEPhI, Moscow, Russia; D.V. Skobeltsyn Institute of Nuclear Physics, M.V. Lomonosov Moscow State University, Moscow, Russia; Fakultät für Physik, Ludwig-Maximilians-Universität München, Munich, Germany; Max-Planck-Institut für Physik (Werner-Heisenberg-Institut), Munich, Germany; Nagasaki Institute of Applied Science, Nagasaki, Japan; Graduate School of Science and Kobayashi-Maskawa Institute, Nagoya University, Nagoya, Japan; INFN Sezione di Napoli, Napoli, Italy; Department of Physics and Astronomy, University of New Mexico, Albuquerque, NM USA; Institute for Mathematics, Astrophysics and Particle Physics, Radboud University Nijmegen/Nikhef, Nijmegen, The Netherlands; Nikhef National Institute for Subatomic Physics and University of Amsterdam, Amsterdam, The Netherlands; Department of Physics, Northern Illinois University, DeKalb, IL USA; Budker Institute of Nuclear Physics, SB RAS, Novosibirsk, Russia; Department of Physics, New York University, New York, NY USA; Ohio State University, Columbus, OH USA; Faculty of Science, Okayama University, Okayama, Japan; Homer L. Dodge Department of Physics and Astronomy, University of Oklahoma, Norman, OK USA; Department of Physics, Oklahoma State University, Stillwater, OK USA; Palacký University, RCPTM, Olomouc, Czech Republic; Center for High Energy Physics, University of Oregon, Eugene, OR USA; LAL, Université Paris-Sud and CNRS/IN2P3, Orsay, France; Graduate School of Science, Osaka University, Osaka, Japan; Department of Physics, University of Oslo, Oslo, Norway; Department of Physics, Oxford University, Oxford, UK; INFN Sezione di Pavia, Pavia, Italy; Department of Physics, University of Pennsylvania, Philadelphia, PA USA; Petersburg Nuclear Physics Institute, Gatchina, Russia; INFN Sezione di Pisa, Pisa, Italy; Department of Physics and Astronomy, University of Pittsburgh, Pittsburgh, PA USA; Laboratorio de Instrumentacao e Fisica Experimental de Particulas-LIP, Lisbon, Portugal; Institute of Physics, Academy of Sciences of the Czech Republic, Praha, Czech Republic; Czech Technical University in Prague, Praha, Czech Republic; Faculty of Mathematics and Physics, Charles University in Prague, Praha, Czech Republic; State Research Center Institute for High Energy Physics, Protvino, Russia; Particle Physics Department, Rutherford Appleton Laboratory, Didcot, UK; Ritsumeikan University, Kusatsu, Shiga Japan; INFN Sezione di Roma, Rome, Italy; INFN Sezione di Roma Tor Vergata, Rome, Italy; INFN Sezione di Roma Tre, Rome, Italy; Faculté des Sciences Ain Chock, Réseau Universitaire de Physique des Hautes Energies-Université Hassan II, Casablanca, Morocco; DSM/IRFU (Institut de Recherches sur les Lois Fondamentales de l’Univers), CEA Saclay (Commissariat à l’Energie Atomique et aux Energies Alternatives), Gif-sur-Yvette, France; Santa Cruz Institute for Particle Physics, University of California Santa Cruz, Santa Cruz, CA USA; Department of Physics, University of Washington, Seattle, WA USA; Department of Physics and Astronomy, University of Sheffield, Sheffield, UK; Department of Physics, Shinshu University, Nagano, Japan; Fachbereich Physik, Universität Siegen, Siegen, Germany; Department of Physics, Simon Fraser University, Burnaby, BC Canada; SLAC National Accelerator Laboratory, Stanford, CA USA; Faculty of Mathematics, Physics and Informatics, Comenius University, Bratislava, Slovak Republic; Department of Physics, University of Cape Town, Cape Town, South Africa; Department of Physics, Stockholm University, Stockholm, Sweden; Physics Department, Royal Institute of Technology, Stockholm, Sweden; Departments of Physics and Astronomy and Chemistry, Stony Brook University, Stony Brook, NY USA; Department of Physics and Astronomy, University of Sussex, Brighton, UK; School of Physics, University of Sydney, Sydney, Australia; Institute of Physics, Academia Sinica, Taipei, Taiwan; Department of Physics, Technion, Israel Institute of Technology, Haifa, Israel; Raymond and Beverly Sackler School of Physics and Astronomy, Tel Aviv University, Tel Aviv, Israel; Department of Physics, Aristotle University of Thessaloniki, Thessaloniki, Greece; International Center for Elementary Particle Physics and Department of Physics, The University of Tokyo, Tokyo, Japan; Graduate School of Science and Technology, Tokyo Metropolitan University, Tokyo, Japan; Department of Physics, Tokyo Institute of Technology, Tokyo, Japan; Department of Physics, University of Toronto, Toronto, ON Canada; TRIUMF, Vancouver, BC Canada; Faculty of Pure and Applied Sciences, University of Tsukuba, Tsukuba, Japan; Department of Physics and Astronomy, Tufts University, Medford, MA USA; Centro de Investigaciones, Universidad Antonio Narino, Bogota, Colombia; Department of Physics and Astronomy, University of California Irvine, Irvine, CA USA; INFN Gruppo Collegato di Udine, Sezione di Trieste, Udine, Italy; Department of Physics, University of Illinois, Urbana, IL USA; Department of Physics and Astronomy, University of Uppsala, Uppsala, Sweden; Instituto de Física Corpuscular (IFIC) and Departamento de Física Atómica, Molecular y Nuclear and Departamento de Ingeniería Electrónica and Instituto de Microelectrónica de Barcelona (IMB-CNM), University of Valencia and CSIC, Valencia, Spain; Department of Physics, University of British Columbia, Vancouver, BC Canada; Department of Physics and Astronomy, University of Victoria, Victoria, BC Canada; Department of Physics, University of Warwick, Coventry, UK; Waseda University, Tokyo, Japan; Department of Particle Physics, The Weizmann Institute of Science, Rehovot, Israel; Department of Physics, University of Wisconsin, Madison, WI USA; Fakultät für Physik und Astronomie, Julius-Maximilians-Universität, Würzburg, Germany; Fachbereich C Physik, Bergische Universität Wuppertal, Wuppertal, Germany; Department of Physics, Yale University, New Haven, CT USA; Yerevan Physics Institute, Yerevan, Armenia; Centre de Calcul de l’Institut National de Physique Nucléaire et de Physique des Particules (IN2P3), Villeurbanne, France; Istanbul Aydin University, Istanbul, Turkey; Division of Physics, TOBB University of Economics and Technology, Ankara, Turkey; Department of Physics, Dogus University, Istanbul, Turkey; Department of Physics Engineering, Gaziantep University, Gaziantep, Turkey; Dipartimento di Fisica e Astronomia, Università di Bologna, Bologna, Italy; Electrical Circuits Department, Federal University of Juiz de Fora (UFJF), Juiz de Fora, Brazil; Federal University of Sao Joao del Rei (UFSJ), Sao Joao del Rei, Brazil; Instituto de Fisica, Universidade de Sao Paulo, Sao Paulo, Brazil; Physics Department, National Institute for Research and Development of Isotopic and Molecular Technologies, Cluj Napoca, Romania; University Politehnica Bucharest, Bucharest, Romania; West University in Timisoara, Timisoara, Romania; Departamento de Física, Universidad Técnica Federico Santa María, Valparaiso, Chile; Department of Modern Physics, University of Science and Technology of China, Anhui, China; Department of Physics, Nanjing University, Jiangsu, China; School of Physics, Shandong University, Shandong, China; Department of Physics and Astronomy, Shanghai Key Laboratory for Particle Physics and Cosmology, Shanghai Jiao Tong University, Shanghai, China; Physics Department, Tsinghua University, Beijing, 100084 China; Dipartimento di Fisica, Università della Calabria, Rende, Italy; Marian Smoluchowski Institute of Physics, Jagiellonian University, Kraków, Poland; Dipartimento di Fisica, Università di Genova, Genoa, Italy; High Energy Physics Institute, Tbilisi State University, Tbilisi, Georgia; Physikalisches Institut, Ruprecht-Karls-Universität Heidelberg, Heidelberg, Germany; ZITI Institut für technische Informatik, Ruprecht-Karls-Universität Heidelberg, Mannheim, Germany; Department of Physics, The University of Hong Kong, Pokfulam, Hong Kong; Department of Physics, The Hong Kong University of Science and Technology, Clear Water Bay, Kowloon Hong Kong, China; Dipartimento di Matematica e Fisica, Università del Salento, Lecce, Italy; Dipartimento di Fisica, Università di Milano, Milan, Italy; Dipartimento di Fisica, Università di Napoli, Napoli, Italy; Dipartimento di Fisica, Università di Pavia, Pavia, Italy; Dipartimento di Fisica E. Fermi, Università di Pisa, Pisa, Italy; Faculdade de Ciências, Universidade de Lisboa, Lisboa, Portugal; Department of Physics, University of Coimbra, Coimbra, Portugal; Centro de Física Nuclear da Universidade de Lisboa, Lisboa, Portugal; Departamento de Fisica, Universidade do Minho, Braga, Portugal; Departamento de Fisica Teorica y del Cosmos and CAFPE, Universidad de Granada, Granada, Spain; Dep Fisica and CEFITEC of Faculdade de Ciencias e Tecnologia, Universidade Nova de Lisboa, Caparica, Portugal; Dipartimento di Fisica, Sapienza Università di Roma, Rome, Italy; Dipartimento di Fisica, Università di Roma Tor Vergata, Rome, Italy; Dipartimento di Matematica e Fisica, Università Roma Tre, Rome, Italy; Centre National de l’Energie des Sciences Techniques Nucleaires, Rabat, Morocco; Faculté des Sciences Semlalia, Université Cadi Ayyad, LPHEA-Marrakech, Marrakech, Morocco; Faculté des Sciences, Université Mohamed Premier and LPTPM, Oujda, Morocco; Faculté des Sciences, Université Mohammed V-Agdal, Rabat, Morocco; Department of Subnuclear Physics, Institute of Experimental Physics of the Slovak Academy of Sciences, Kosice, Slovak Republic; Department of Physics, University of Johannesburg, Johannesburg, South Africa; School of Physics, University of the Witwatersrand, Johannesburg, South Africa; The Oskar Klein Centre, Stockholm, Sweden; Department of Physics and Astronomy, York University, Toronto, ON Canada; ICTP, Trieste, Italy; Dipartimento di Chimica, Fisica e Ambiente, Università di Udine, Udine, Italy; CERN, 1211 Geneva 23, Switzerland

## Abstract

A search is presented for the direct pair production of a chargino and a neutralino $pp\rightarrow \tilde{\chi }_1^\pm \tilde{\chi }_2^0$, where the chargino decays to the lightest neutralino and the $W$ boson, $\tilde{\chi }_1^\pm \rightarrow \tilde{\chi }_1^0(W^{\pm }\rightarrow \ell ^{\pm }\nu )$, while the neutralino decays to the lightest neutralino and the 125 GeV Higgs boson, $\tilde{\chi }_2^0\rightarrow \tilde{\chi }_1^0(h\rightarrow bb/\gamma \gamma /\ell ^{\pm }\nu qq)$. The final states considered for the search have large missing transverse momentum, an isolated electron or muon, and one of the following: either two jets identified as originating from bottom quarks, or two photons, or a second electron or muon with the same electric charge. The analysis is based on 20.3 $\mathrm {fb}^{-1}$ of $\sqrt{s}=8{\mathrm {\ TeV}}$ proton–proton collision data delivered by the Large Hadron Collider and recorded with the ATLAS detector. Observations are consistent with the Standard Model expectations, and limits are set in the context of a simplified supersymmetric model.

## Introduction

Supersymmetry (SUSY) [1–9] proposes the existence of new particles with spin differing by one half unit from that of their Standard Model (SM) partners. In the Minimal Supersymmetric Standard Model (MSSM) [10–14], charginos, $\tilde{\chi }_{1,2}^\pm$, and neutralinos, $\tilde{\chi }_{1,2,3,4}^0$, are the mass-ordered eigenstates formed from the linear superposition of the SUSY partners of the Higgs and electroweak gauge bosons (higgsinos, winos and bino). In $R$-parity-conserving models, SUSY particles are pair-produced in colliders and the lightest SUSY particle (LSP) is stable. In many models the LSP is assumed to be a bino-like $\tilde{\chi }_1^0$, which is weakly interacting. Naturalness arguments [15, 16] suggest that the lightest of the charginos and neutralinos may have masses at the electroweak scale, and may be accessible at the Large Hadron Collider (LHC) [17]. Furthermore, direct pair production of charginos and neutralinos may be the dominant production of supersymmetric particles if the superpartners of the gluon and quarks are heavier than a few TeV.

In SUSY scenarios where the masses of the pseudoscalar Higgs boson and the superpartners of the leptons are larger than those of the produced chargino and neutralino, the chargino decays to the lightest neutralino and the $W$ boson, while the next-to-lightest neutralino decays to the lightest neutralino and the SM-like Higgs or $Z$ boson. This paper focuses on SUSY scenarios where the decay to the Higgs boson is the dominant one. This happens when the mass splitting between the two lightest neutralinos is larger than the Higgs boson mass and the higgsinos are much heavier than the winos, causing the composition of the lightest chargino and next-to-lightest neutralino to be wino-like and nearly mass degenerate.

A simplified SUSY model [18, 19] is considered for the optimisation of the search and the interpretation of results. It describes the direct production of $\tilde{\chi }_1^\pm$ and $\tilde{\chi }_2^0$, where the masses and the decay modes of the relevant particles ($\tilde{\chi }_1^\pm$, $\tilde{\chi }_1^0$, $\tilde{\chi }_2^0$) are the only free parameters. It is assumed that the $\tilde{\chi }_1^\pm$ and $\tilde{\chi }_2^0$ are pure wino states and degenerate in mass, while the $\tilde{\chi }_1^0$ is a pure bino state. The prompt decays $\tilde{\chi }_1^\pm \rightarrow W^\pm \tilde{\chi }_1^0$ and $\tilde{\chi }_2^0\rightarrow h\tilde{\chi }_1^0$ are assumed to have 100 % branching fractions. The Higgs boson mass is set to $125{\mathrm {\ GeV}}$, which is consistent with the measured value [20], and its branching fractions are assumed to be the same as in the SM. The latter assumption is motivated by those SUSY models in which the mass of the pseudoscalar Higgs boson is much larger than the $Z$ boson mass.

**Fig. 1:**
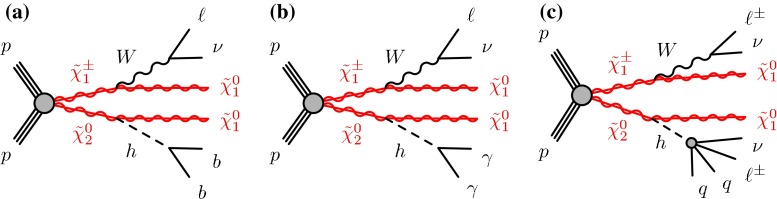
Diagrams for the direct pair production of $\tilde{\chi }_1^\pm \tilde{\chi }_2^0$ and the three decay modes studied in this paper. For the same-sign dilepton channel (c), only the dominant decay mode is shown. **a** One lepton and two $b$-quarks channel, **b** one lepton and two photons channel and **c** same-sign dilepton channel

The search presented in this paper targets leptonic decays of the $W$ boson and three Higgs boson decay modes as illustrated in Fig. 1. The Higgs boson decays into a pair of $b$-quarks, or a pair of photons, or a pair of $W$ bosons where at least one of the bosons decays leptonically. The final states therefore contain missing transverse momentum from neutrinos and neutralinos, one lepton ($\ell =e$ or $\mu$), and one of the following: two $b$-quarks ($\ell {}bb$), or two photons ($\ell \gamma \gamma$), or an additional lepton with the same electric charge ($\ell ^{\pm }\ell ^{\pm }$). The Higgs boson candidate can be fully reconstructed with the $\ell {}bb$ and $\ell \gamma \gamma$ signatures. The $\ell ^{\pm }\ell ^{\pm }$ signature does not allow for such reconstruction and it is considered because of its small SM background. Its main signal contribution is due to $h\rightarrow {}WW$, with smaller contributions from $h\rightarrow {}ZZ$ and $h\rightarrow {}\tau \tau$ when some of the visible decay products are missed during the event reconstruction.

The analysis is based on 20.3 $\mathrm {fb}^{-1}$ of $\sqrt{s}=8{\mathrm {\ TeV}}$ proton–proton collision data delivered by the LHC and recorded with the ATLAS detector. Previous searches for charginos and neutralinos at the LHC have been reported by the ATLAS [21–23] and CMS [24, 25] collaborations. Similar searches were conducted at the Tevatron [26, 27] and LEP [28–32].

The results of this paper are combined with those of the ATLAS search using the three-lepton and missing transverse momentum final state, performed with the same dataset [21]. The three-lepton selections may contain up to two hadronically decaying $\tau {}$ leptons, providing sensitivity to the $h\rightarrow \tau \tau /WW/ZZ$ Higgs boson decay modes. The statistical combination of the results is facilitated by the fact that all event selections were constructed not to overlap.

This paper is organised in the following way: the ATLAS detector is briefly described in Sect. 2, followed by a description of the Monte Carlo simulation in Sect. 3. In Sect. 4 the common aspects of the event reconstruction are illustrated; Sects. 5, 6, and 7 describe the channel-specific features; Sect. 8 discusses the systematic uncertainties; the results and conclusions are presented in Sects. 9 and 10.

## The ATLAS detector

ATLAS is a multipurpose particle physics experiment [33]. It consists of detectors forming a forward-backward symmetric cylindrical geometry.^1^ The inner detector (ID) covers $$|\eta |<2.5$$ and consists of a silicon pixel detector, a semiconductor microstrip tracker, and a transition radiation tracker. The ID is surrounded by a thin superconducting solenoid providing a $$2\,\mathrm{T}$$ axial magnetic field. A high-granularity lead/liquid-argon (LAr) sampling calorimeter measures the energy and the position of electromagnetic showers within $$|\eta |<3.2$$. Sampling calorimeters with LAr are also used to measure hadronic showers in the endcap (1.5 $$<$$$$|\eta |$$$$<$$ 3.2) and forward (3.1 $$<$$$$|\eta |$$$$<$$ 4.9) regions, while a steel/scintillator tile calorimeter measures hadronic showers in the central region ($$|\eta |$$$$<$$ 1.7). The muon spectrometer (MS) surrounds the calorimeters and consists of three large superconducting air-core toroid magnets, each with eight coils, precision tracking chambers ($$|\eta |$$$$<$$ 2.7), and fast trigger chambers ($$|\eta |$$$$<$$ 2.4). A three-level trigger system selects events to be recorded for permanent storage.

## Monte Carlo simulation

**Table 1:** Simulated samples used for background estimates. “Tune” refers to the choice of parameters used for the underlying-event generation

Process	Generator	Cross section	Tune	PDF set
Single top, $t$-channel	AcerMC [34] $+$ Pythia6 [35]	NNLO $+$ NNLL [36]	AUET2B [37]	CTEQ6L1 [38]
Single top, $s$-channel	Powheg [39, 40] $+$ Pythia6	NNLO $+$ NNLL [41]	Perugia2011C [42]	CT10 [43]
$tW$	Powheg + Pythia6	NNLO $+$ NNLL [44]	Perugia2011C	CT10
$t\bar{t}$	Powheg + Pythia6	NNLO $+$ NNLL [45–50]	Perugia2011C	CT10
$t\bar{t}W$, $t\bar{t}Z$	MadGraph [51] $+$ Pythia6	NLO	AUET2B	CTEQ6L1
$W$, $Z$ ($\ell {}bb$ channel)	Sherpa [52]	NLO	–	CT10
$W$, $Z$ ($\ell ^{\pm }\ell ^{\pm }$ channel)	Alpgen [53] $+$ Pythia6	NLO	Perugia2011C	CTEQ6L1
$WW$, $WZ$, $ZZ$	Sherpa	NLO	–	CT10
$W\gamma$ $W\gamma \gamma$	Alpgen $+$ Pythia6	NLO	AUET2B	CTEQ6L1
$Z\gamma$, $Z\gamma \gamma$	Sherpa	NLO	–	CT10
$Wh$, $Zh$	Pythia8 [54]	NNLO (QCD) $+$ NLO (EW) [55]	AU2 [56]	CTEQ6L1
$t\bar{t}h$	Pythia8	NLO (QCD) [55]	AU2	CTEQ6L1

The event generators, the accuracy of theoretical cross sections, the underlying-event parameter tunes, and the parton distribution function (PDF) sets used for simulating the SM background processes are summarised in Table 1.

The SUSY signal samples are produced with Herwig++  [57] using the CTEQ6L1 PDF set. Signal cross sections are calculated at next-to-leading order (NLO) in the strong coupling constant using Prospino2  [58]. These agree with the NLO calculations matched to resummation at next-to-leading-logarithmic (NLL) accuracy within $\sim$2 % [59, 60]. For each cross section, the nominal value and its uncertainty are taken respectively from the centre and the spread of the cross-section predictions using different PDF sets and their associated uncertainties, as well as from variations of factorisation and renormalisation scales, as described in Ref. [61].

The propagation of particles through the ATLAS detector is modelled with GEANT4  [62] using the full ATLAS detector simulation [63] for all Monte Carlo (MC) simulated samples, except for $t\bar{t}$ production and the SUSY signal samples in which the Higgs boson decays to two $b$-quarks, for which a fast simulation based on a parametric response of the electromagnetic and hadronic calorimeters is used [64]. The effect of multiple proton–proton collisions in the same or nearby beam bunch crossings (in-time or out-of-time pile-up) is incorporated into the simulation by overlaying additional minimum-bias events generated with Pythia6 onto hard-scatter events. Simulated events are weighted so that the distribution of the average number of interactions per bunch crossing matches that observed in data, but are otherwise reconstructed in the same manner as data.

## Event reconstruction

The data sample considered in this analysis was collected with a combination of single-lepton, dilepton, and diphoton triggers. After applying beam, detector, and data-quality requirements, the dataset corresponds to an integrated luminosity of 20.3 $\mathrm {fb}^{-1}$, with an uncertainty of 2.8 % derived following the methodology detailed in Ref. [65].

Vertices compatible with the proton-proton interactions are reconstructed using tracks from the ID. Events are analysed if the primary vertex has five or more tracks, each with transverse momentum $p_\mathrm {T}>400{\mathrm {\ MeV}}$, unless stated otherwise. The primary vertex of an event is identified as the vertex with the largest $\sum p_\mathrm {T}^2$ of the associated tracks.

Electron candidates are reconstructed from calibrated clustered energy deposits in the electromagnetic calorimeter and a matched ID track, which in turn determine the $p_\mathrm {T}$ and $\eta$ of the candidates respectively. Electrons must satisfy “medium” cut-based identification criteria, following Ref. [66], and are required to have $p_\mathrm {T}>10{\mathrm {\ GeV}}$ and $|\eta |<2.47$.

Muon candidates are reconstructed by combining tracks in the ID and tracks or segments in the MS [67] and are required to have $p_\mathrm {T}>10{\mathrm {\ GeV}}$ and $|\eta |<2.5$. To suppress cosmic-ray muon background, events are rejected if they contain a muon having transverse impact parameter with respect to the primary vertex $|d_0|>0.2$ mm or longitudinal impact parameter with respect to the primary vertex $|z_0|>1$ mm.

Photon candidates are reconstructed from clusters of energy deposits in the electromagnetic calorimeter. Clusters without matching tracks as well as those matching one or two tracks consistent with a photon conversion are considered. The shape of the cluster must match that expected for an electromagnetic shower, using criteria tuned for robustness under the pile-up conditions of 2012 [68]. The cluster energy is calibrated separately for converted and unconverted photon candidates using simulation. In addition, $\eta$-dependent correction factors determined from $Z\rightarrow e^+e^-$ events are applied to the cluster energy, as described in Ref. [68]. The photon candidates must have $p_\mathrm {T}>20{\mathrm {\ GeV}}$ and $|\eta |<2.37$, excluding the transition region $1.37<|\eta |<1.56$ between the central and endcap electromagnetic calorimeters. The tighter $\eta$ requirement on photons, as compared to electrons, reflects the poorer photon resolution in the transition region and for $2.37\le |\eta |<2.47$.

Jets are reconstructed with the anti-$k_t$ algorithm [69] with a radius parameter of 0.4 using three-dimensional clusters of energy in the calorimeter [70] as input. The clusters are calibrated, weighting differently the energy deposits arising from the electromagnetic and hadronic components of the showers. The final jet energy calibration corrects the calorimeter response to the particle-level jet energy [71, 72]; the correction factors are obtained from simulation and then refined and validated using data. Corrections for in-time and out-of-time pile-up are also applied, as described in Ref. [73]. Events containing jets failing to meet the quality criteria described in Ref. [71] are rejected to suppress non-collision background and events with large noise in the calorimeters.

Jets with $p_\mathrm {T}>20{\mathrm {\ GeV}}$ are considered in the central pseudorapidity ($|\eta |<2.4$) region, and jet $p_\mathrm {T}>30{\mathrm {\ GeV}}$ is required in the forward ($2.4<|\eta |<4.5$) region. For central jets, the $p_\mathrm {T}$ threshold is lower since it is possible to suppress pile-up using information from the ID, the “jet vertex fraction” (JVF). This is defined as the $p_\mathrm {T}$-weighted fraction of tracks within the jet that originate from the primary vertex of the event, and is $-1$ if there are no tracks within the jet. Central jets can also be tagged as originating from bottom quarks (referred to as $b$-jets) using the MV1 multivariate $b$-tagging algorithm based on quantities related to impact parameters of tracks and reconstructed secondary vertices [74]. The efficiency of the $b$-tagging algorithm depends on the operating point chosen for each channel, and is reported in Sects. 5 and 7.

Hadronically decaying $\tau$ leptons are reconstructed as 1- or 3-prong hadronic jets within $|\eta |<2.47$, and are required to have $p_\mathrm {T}>20{\mathrm {\ GeV}}$ after being calibrated to the $\tau$ energy scale [75]. Final states with hadronically decaying $\tau$ leptons are not considered here; however, identified $\tau$ leptons are used in the overlap removal procedure described below, as well as to ensure that the same-sign lepton channel does not overlap with the three-lepton search [21] that is included in the combined result.

Potential ambiguities between candidate leptons, photons and jets are resolved by removing one or both objects if they are separated by $\Delta {}R\equiv \sqrt{(\Delta \phi )^2+(\Delta \eta )^2}$ below a threshold. This process eliminates duplicate objects reconstructed from a single particle, and suppresses leptons and photons contained inside hadronic jets. The thresholds and the order in which overlapping objects are removed are summarised in Table 2. In the same-sign channel, $e^+e^-$ and $\mu ^+\mu ^-$ pairs with $m_{\ell ^+\ell ^-}<12~{\mathrm {\ GeV}}{}$ are also removed. The remaining leptons and photons are referred to as “preselected” objects.

**Table 2:** Summary of the overlap removal procedure. Potential ambiguities are resolved by removing nearby objects in the indicated order, from top to bottom. Different $\Delta {}R$ separation requirements are used in the three channels

Candidates	$\Delta {}R$ threshold			Candidate removed
	$\ell {}bb$	$\ell \gamma \gamma$	$\ell ^{\pm }\ell ^{\pm }$	
$e$–$e$	0.1	–	0.05	Lowest-$p_\mathrm {T}$ $e$
$e$–$\gamma$	–	0.4	–	$e$
Jet–$\gamma$	–	0.4	–	Jet
Jet–$e$	0.2	0.2	0.2	Jet
$\tau {}$–$e$ or $\tau {}$–$\mu {}$	–	–	0.2	$\tau$
$\mu$–$\gamma$	–	0.4	–	$\mu$
$e$–jet or $\mu$–jet	0.4	0.4	0.4	$e$ or $\mu$
$e$–$\mu$	$0.1$	–	$0.1$	Both
$\mu$–$\mu$	$0.05$	–	$0.05$	Both
Jet–$\tau$	–	–	0.2	Jet

Isolation criteria are applied to improve the purity of reconstructed objects. The criteria are based on the scalar sum of the transverse energies $E_\mathrm {T}$ of the calorimeter cell clusters within a radius $\Delta {}R$ of the object ($E_\mathrm {T}^{\mathrm {cone}\Delta {}R}$), and on the scalar sum of the $p_\mathrm {T}$ of the tracks within $\Delta {}R$ and associated with the primary vertex ($p_\mathrm {T}^{\mathrm {cone}\Delta {}R}$). The contribution due to the object itself is not included in either sum. The values used in the isolation criteria depend on the channel; they are specified in Sects. 5, 6 and 7.

The missing transverse momentum, $\mathbf {p}_\mathrm {T}^\mathrm {\,miss}$ (with magnitude $E_{\mathrm {T}}^{\mathrm {miss}}$), is the negative vector sum of the transverse momenta of all preselected electrons, muons, and photons, as well as jets and calorimeter energy clusters with $|\eta |$ $<$ 4.9 not associated with these objects. Clusters that are associated with electrons, photons and jets are calibrated to the scale of the corresponding objects [76, 77].

The efficiencies for electrons, muons, and photons to satisfy the reconstruction and identification criteria are measured in control samples, and corrections are applied to the simulated samples to reproduce the efficiencies in data. Similar corrections are also applied to the trigger efficiencies, as well as to the jet $b$-tagging efficiency and misidentification probability.

## One lepton and two $b$-jets channel

**Table 3:** Selection requirements for the signal, control and validation regions of the one lepton and two $b$-jets channel. The number of leptons, jets, and $b$-jets is labelled with $n_\mathrm {lepton}$, $n_\mathrm {jet}$, and $n_{b-\mathrm {jet}}$respectively

	SR$\ell bb$-1	SR$\ell bb$-2	CR$\ell bb$-T	CR$\ell bb$-W	VR$\ell bb$-1	VR$\ell bb$-2
$n_\mathrm {lepton}$	1	1	1	1	1	1
$n_\mathrm {jet}$	2–3	2–3	2–3	2	2–3	2–3
$n_{b-\mathrm {jet}}$	2	2	2	1	2	2
$E_{\mathrm {T}}^{\mathrm {miss}}$ (GeV)	$>$100	$>$100	$>$100	$>$100	$>$100	$>$100
$m_\mathrm {CT}$ (GeV)	$>$160	$>$160	100–160	$>$160	100–160	$>$160
$m_\mathrm {T}^W$ (GeV)	100–130	$>$130	$>$100	$>$40	40–100	40–100

### Event selection

The events considered in the one lepton and two $b$-jets channel are recorded with a combination of single-lepton triggers with a $p_\mathrm {T}$ threshold of 24 GeV. To ensure that the event is triggered with a constant high efficiency, the offline event selection requires exactly one signal lepton ($e$ or $\mu$) with $p_\mathrm {T}>25{\mathrm {\ GeV}}$. The signal electrons must satisfy the “tight” identification criteria of Ref. [66], as well as $|d_0|/\sigma _{d_0}<5$, where $\sigma _{d_0}$ is the error on $d_0$, and $|z_0\sin \theta |<0.4$ mm. The signal muons must satisfy $|\eta |<2.4$, $|d_0|/\sigma _{d_0}<3$, and $|z_0\sin \theta |<0.4$ mm. The signal electrons (muons) are required to satisfy the isolation criteria $E_\mathrm {T}^{\mathrm {cone}0.3}/p_\mathrm {T}<0.18$ (0.12) and $p_\mathrm {T}^{\mathrm {cone}0.3}/p_\mathrm {T}<0.16$ (0.12).

Events with two or three jets are selected, and the jets can be either central ($|\eta |<2.4$) or forward ($2.4<|\eta |<4.9$). Central jets have $p_\mathrm {T}>25~{\mathrm {\ GeV}}$, and forward jets have $p_\mathrm {T}>30~{\mathrm {\ GeV}}$. For central jets with $p_\mathrm {T}<50{\mathrm {\ GeV}}$, the $\mathrm {JVF}{}$ must be $>0.5$. Events must contain exactly two $b$-jets and these must be the highest-$p_\mathrm {T}$ central jets. The chosen operating point of the $b$-tagging algorithm identifies $b$-jets in simulated $t\bar{t}$ events with an efficiency of 70 %; it misidentifies charm jets 20 % of the time and light-flavour (including gluon-induced) jets less than $1\,\%$ of the time.

After the requirement of $E_{\mathrm {T}}^{\mathrm {miss}}>100$ GeV, the dominant background contributions in the $\ell {}bb$ channel are $t\bar{t}$, $W+\mathrm {jets}$, and single-top $Wt$ production. Their contributions are suppressed using the kinematic selections described below, which define the two signal regions (SR) SR$\ell bb$-1 and SR$\ell bb$-2 summarised in Table 3.

The contransverse mass $m_\mathrm {CT}$  [78, 79] is defined as

1 $$\begin{aligned} m_\mathrm {CT} = \sqrt{(E_\mathrm {T}^{b_1}+E_\mathrm {T}^{b_2})^2 - |\mathbf {p}_\mathrm {T}^{b_1}-\mathbf {p}_\mathrm {T}^{b_2}|^2}, \end{aligned}$$

where $E_\mathrm {T}^{b_i}$ and $\mathbf {p}_\mathrm {T}^{b_i}$ are the transverse energy and momentum of the $i$th $b$-jet. The SM $t\bar{t}$ background has an upper endpoint at $m_\mathrm {CT}$ of approximately $m_t$, and is efficiently suppressed by requiring $m_\mathrm {CT} >160{\mathrm {\ GeV}}$.

The transverse mass $m_\mathrm {T}^W$, describing $W$ candidates in background events, is defined as

2 $$\begin{aligned} m_\mathrm {T}^W= \sqrt{2 E_\mathrm {T}^\ell E_{\mathrm {T}}^{\mathrm {miss}}- 2 \mathbf {p}_\mathrm {T}^\ell \cdot \mathbf {p}_\mathrm {T}^\mathrm {miss}}, \end{aligned}$$

where $E_\mathrm {T}^\ell$ and $\mathbf {p}_\mathrm {T}^\ell$ are the transverse energy and momentum of the lepton. Requiring $m_\mathrm {T}^W>100{\mathrm {\ GeV}}$ efficiently suppresses the $W$ $+$ jets background. The two SRs are distinguished by requiring $100<m_\mathrm {T}^W<130{\mathrm {\ GeV}}$ for SR$\ell bb$-1 and $m_\mathrm {T}^W>130{\mathrm {\ GeV}}$ for SR$\ell bb$-2. The first signal region provides sensitivity to signal models with a mass splitting between $\tilde{\chi }_1^0$ and $\tilde{\chi }_2^0$ similar to the Higgs boson mass, while the second one targets larger mass splittings.

In each SR, events are classified into five bins of the invariant mass $m_{bb}$ of the two $b$-jets as 45–75–105–135–165–195 GeV. In the SRs, about 70 % of the signal events due to $h\rightarrow b\bar{b}$ populate the central bin of 105–135 GeV. The other four bins (sidebands) are used to constrain the background normalisation, as described below.

**Fig. 2:**
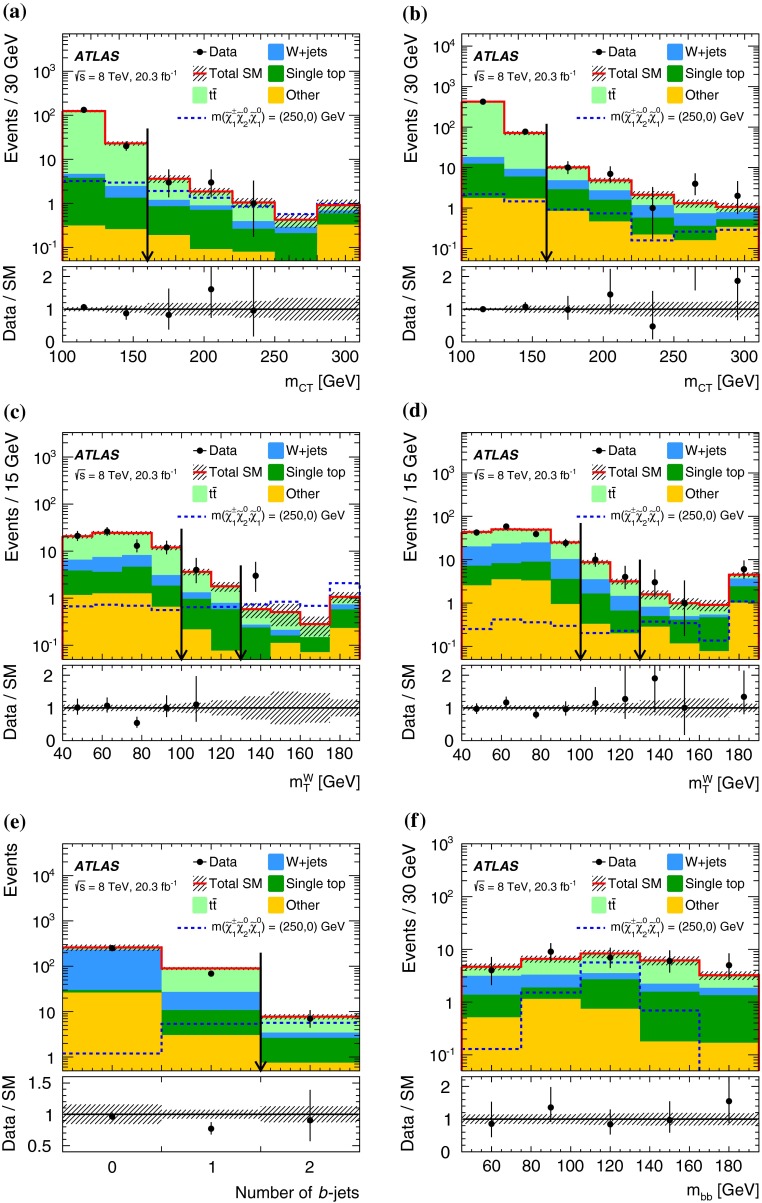
Distributions of contransverse mass $m_\mathrm {CT}$, transverse mass of the $W$-candidate $m_\mathrm {T}^W$, number of $b$-jets, and invariant mass of the $b$-jets $m_{bb}$ for the one lepton and two $b$-jets channel in the indicated regions. The *stacked background histograms* are obtained from the background-only fit. The *hashed areas* represent the total uncertainties on the background estimates after the fit. The rightmost bins in **a**–**d** include overflow. The distributions of a signal hypothesis are also shown without stacking on the background histograms. The *vertical arrows* indicate the boundaries of the signal regions. The *lower panels* show the ratio of the data to the SM background prediction. **a** $m_\mathrm {CT}$ in CR$\ell bb$-T, SR$\ell bb$-1 and SR$\ell bb$-2, central $m_{bb}$ bin, **b** $m_\mathrm {CT}$ in CR$\ell bb$-T, SR$\ell bb$-1 and SR$\ell bb$-2, $m_{bb}$ sidebands, **c** $m_\mathrm {T}^W$ in VR$\ell bb$-2, SR$\ell bb$-1 and SR$\ell bb$-2, central $m_{bb}$ bin, **d** $m_\mathrm {T}^W$ in VR$\ell bb$-2, SR$\ell bb$-1 and SR$\ell bb$-2, $m_{bb}$ sidebands, **e** number of $b$-jets in SR$\ell bb$-1 and SR$\ell bb$-2 without the $b$-jet multiplicity requirement, central $m_{bb}$ bin, **f** $m_{bb}$ in SR$\ell bb$-1 and SR$\ell bb$-2

**Table 4:** Event yields and SM expectation in the one lepton and two $b$-jets channel obtained with the background-only fit. “Other” includes $Z$ $+$ jets, $WW$, $WZ$, $ZZ$, $Zh$ and $Wh$ processes. The errors shown include statistical and systematic uncertainties

	SR$\ell bb$-1$105<m_{bb} <135$ GeV	SR$\ell bb$-2$m_{bb}$ sidebands	SR$\ell bb$-1	SR$\ell bb$-2	CR$\ell bb$-T	CR$\ell bb$-W	VR$\ell bb$-1	VR$\ell bb$-2
Observed events	4	3	14	10	651	1547	885	235
SM expectation	6.0 $\pm$ 1.3	2.8 $\pm$ 0.8	13.1 $\pm$ 2.4	8.8 $\pm$ 1.7	642 $\pm$ 25	1560 $\pm$ 40	880 $\pm$ 90	245 $\pm$ 17
$t\bar{t}$	3.8 $\pm$ 1.2	1.4 $\pm$ 0.7	8.0 $\pm$ 2.4	3.1 $\pm$ 1.4	607 $\pm$ 25	680 $\pm$ 60	680 $\pm$ 90	141 $\pm$ 18
$W$ + jets	0.6 $\pm$ 0.3	0.2 $\pm$ 0.1	2.7 $\pm$ 0.5	1.7 $\pm$ 0.3	11 $\pm$ 2	690 $\pm$ 60	99 $\pm$ 12	62 $\pm$ 8
Single top	1.3 $\pm$ 0.4	0.7 $\pm$ 0.4	1.9 $\pm$ 0.6	2.5 $\pm$ 1.1	20 $\pm$ 4	111 $\pm$ 14	80 $\pm$ 10	27 $\pm$ 4
Other	0.3 $\pm$ 0.1	0.5 $\pm$ 0.1	0.5 $\pm$ 0.1	1.5 $\pm$ 0.2	4 $\pm$ 1	76 $\pm$ 8	16 $\pm$ 2	15 $\pm$ 1

### Background estimation

The contributions from the $$t\bar{t}$$ and $$W+\mathrm {jets}$$ background sources are estimated from simulation, and normalised to data in dedicated control regions defined in the following paragraphs. The contribution from multi-jet production, where the signal lepton is a misidentified jet or comes from a heavy-flavour hadron decay or photon conversion, is estimated using the “matrix method” described in Ref. [22], and is found to be less than 3 % of the total background in all regions and is thus neglected. The remaining sources of background (single top, $$Z$$$$+$$ jets, $$WW$$, $$WZ$$, $$ZZ$$, $$Zh$$ and $$Wh$$ production) are estimated from simulation.

Two control regions (CR), CR$\ell bb$-T and CR$\ell bb$-W, are designed to constrain the normalisations of the $t\bar{t}$ and $W+\mathrm {jets}$ backgrounds respectively. The acceptance for $t\bar{t}$ events is increased in CR$\ell bb$-T by modifying the requirement on $m_\mathrm {CT}$ to $100<m_\mathrm {CT} <160{\mathrm {\ GeV}}$. The acceptance of $W+\mathrm {jets}$ events is increased in CR$\ell bb$-W by requiring $m_\mathrm {T}^W{}>40{\mathrm {\ GeV}}$ and exactly two jets, of which only one is $b$-tagged. These two control regions are summarised in Table 3. The control regions are defined to be similar to the signal regions in order to reduce systematic uncertainties on the extrapolation to the signal regions; at the same time they are dominated by the targeted background processes and the expected contamination by signal is small.

As in the signal regions, the control regions are binned in $m_{bb}$ ($m_{bj}$ in the case of CR$\ell bb$-W). A “background-only” likelihood fit is performed, in which the predictions of the simulated background processes without any signal hypothesis are fit simultaneously to the data yields in eight $m_{bb}$ sideband bins of the SRs and the ten $m_{bb}$ bins of the CRs. This fit, as well as the limit-setting procedure, is performed using the HistFitter package described in Ref. [80]. The two free parameters of the fit, namely the normalisations of the $t\bar{t}$ and $W+\mathrm {jets}$ background components, are constrained by the number of events observed in the control regions and signal region sidebands, where the number of events is described by a Poisson probability density function. The remaining nuisance parameters correspond to the sources of systematic uncertainty described in Sect. 8. They are taken into account with their uncertainties, and adjusted to maximise the likelihood. The yields estimated with the background-only fit are reported in Table 4, as well as the resulting predictions in SR$\ell bb$-1 and SR$\ell bb$-2 for $105<m_{bb} <135$ GeV. While CR$\ell bb$-T is dominated by $t\bar{t}$ events, CR$\ell bb$-W is populated evenly by $t\bar{t}$ and $W+\mathrm {jets}$ events, which causes the normalisations of the $t\bar{t}$ and $W+\mathrm {jets}$ contributions to be negatively correlated after the fit. As a result, the uncertainties on individual background sources do not add up quadratically to the uncertainty on the total SM expectation. The normalisation factors are found to be $1.03\pm 0.15$ for $t\bar{t}$ and $0.79\pm 0.07$ for $W+\mathrm {jets}$, where the errors include statistical and systematic uncertainties.

To validate the background modelling, two validation regions (VR) are defined similarly to the SRs except for requiring $40<m_\mathrm {T}^W<100{\mathrm {\ GeV}}$, and requiring $100<m_\mathrm {CT} <160{\mathrm {\ GeV}}$ for VR$\ell bb$-1 and $m_\mathrm {CT} >160{\mathrm {\ GeV}}$ for VR$\ell bb$-2 as summarised in Table 3. The yields in the VRs are shown in Table 4 after the background-only fit, which does not use the data in the VRs to constrain the background. The data event yields are found to be consistent with background expectations. Figure 2 shows the data distributions of $m_\mathrm {CT}$, $m_\mathrm {T}^W$, $n_{b-\mathrm {jet}}$ and $m_{bb}$ compared to the SM expectations in various regions. The data agree well with the SM expectations in all distributions.

## One lepton and two photons channel

### Event selection

Events recorded with diphoton or single-lepton triggers are used in the one lepton and two photons channel. For the diphoton trigger, the transverse momentum thresholds at trigger level for the highest-$p_\mathrm {T}$ (leading) and second highest-$p_\mathrm {T}$ (sub-leading) photons are $35{\mathrm {\ GeV}}$ and $25{\mathrm {\ GeV}}$ respectively. For these events, the event selection requires exactly one signal lepton ($e$ or $\mu$) and exactly two signal photons, with $p_\mathrm {T}$ thresholds of $15{\mathrm {\ GeV}}$ for electrons, $10{\mathrm {\ GeV}}$ for muons, and 40 (27) GeV for leading (sub-leading) photons. In addition, events recorded with single-lepton triggers, which have transverse momentum thresholds at trigger level of 24 GeV, are used. For these events, the selection requires $p_\mathrm {T}$ thresholds of $25{\mathrm {\ GeV}}$ for electrons and muons, and 40 (20) GeV for leading (sub-leading) photons.

**Table 5:** Selection requirements for the signal and validation regions of the one lepton and two photons channel. The number of leptons and photons is labelled with $n_\mathrm {lepton}$ and $n_{\gamma }$ respectively

	SR$\ell \gamma \gamma$-1	SR$\ell \gamma \gamma$-2	VR$\ell \gamma \gamma$-1	VR$\ell \gamma \gamma$-2
$n_\mathrm {lepton}$	1	1	1	1
$n_{\gamma }$	2	2	2	2
$E_{\mathrm {T}}^{\mathrm {miss}}$ (GeV)	$>$40	$>$40	$<$40	–
$\Delta \phi (W,h)$	$>$2.25	$>$2.25	–	$<$2.25
$m_{\mathrm {T}}^{{W\!\gamma _1}}$(GeV)	$>$150	$<$150		
	and	or	–	–
$$m_{\mathrm {T}}^{{W\!\gamma _2}}$$(GeV)	$$> $$80	$$< $$80		

**Fig. 3:**
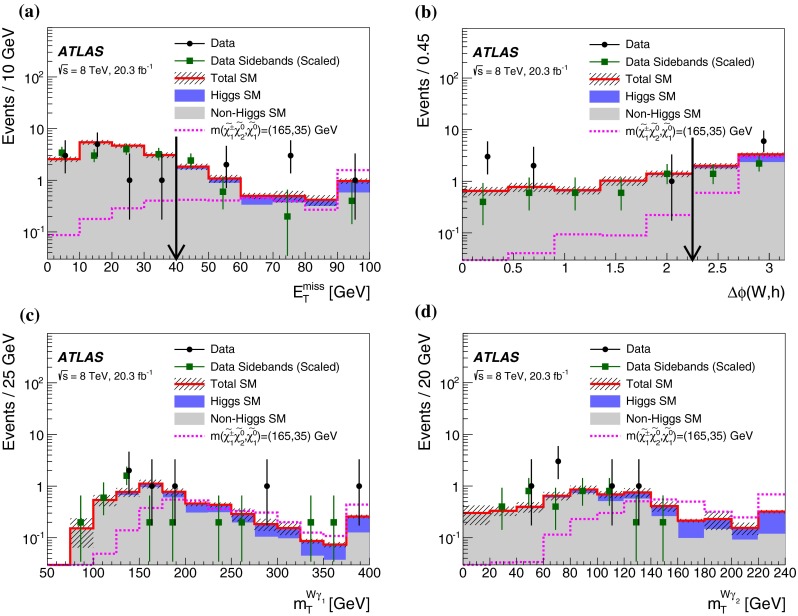
Distributions of missing transverse momentum $E_{\mathrm {T}}^{\mathrm {miss}}$, azimuth difference between the $W$ and Higgs boson candidates $\Delta \phi (W,h)$, transverse mass of the $W$ and photon system $m_{\mathrm {T}}^{{W\!\gamma _1}}$ and $m_{\mathrm {T}}^{{W\!\gamma _2}}$ in the one lepton and two photons signal regions for the Higgs-mass window ($120<m_{\gamma \gamma }<130{\mathrm {\ GeV}}$). The *vertical arrows* indicate the boundaries of the signal regions. The *filled and hashed* *areas* represent the stacked histograms of the simulation-based background cross check and the total uncertainties. The contributions from non-Higgs backgrounds are scaled by 10 GeV/50 GeV $=$ 0.2 from the $m_{\gamma \gamma }$ sideband ($100<m_{\gamma \gamma }<120{\mathrm {\ GeV}}$ and $130<m_{\gamma \gamma }<160{\mathrm {\ GeV}}$) into the Higgs-mass window. The rightmost bins in **a**, **c**, and **d** include overflow. Scaled data in the sideband are shown as *squares*, while events in the Higgs-mass window are shown as *circles*. The distributions of a signal hypothesis are also shown *without stacking* on the background histograms. **a** $E_{\mathrm {T}}^{\mathrm {miss}}$ in SR$\ell \gamma \gamma$-1 and SR$\ell \gamma \gamma$-2 without $E_{\mathrm {T}}^{\mathrm {miss}}$ cut, **b** $\Delta \phi (W,h)$ in SR$\ell \gamma \gamma$-1 and SR$\ell \gamma \gamma$-2 without $\Delta \phi (W,h)$ cut, **c** $m_{\mathrm {T}}^{{W\!\gamma _1}}$ in SR$\ell \gamma \gamma$-1 and SR$\ell \gamma \gamma$-2 without $m_{\mathrm {T}}^{{W\!\gamma _i}}$ cuts, **d** $m_{\mathrm {T}}^{{W\!\gamma _2}}$ in SR$\ell \gamma \gamma$-1 and SR$\ell \gamma \gamma$-2 without $m_{\mathrm {T}}^{{W\!\gamma _i}}$ cuts

**Fig. 4:**
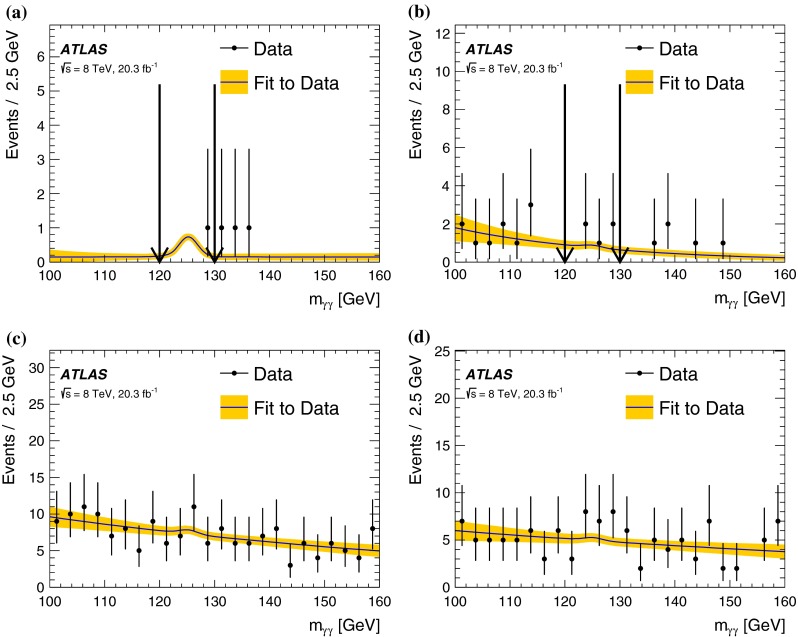
Results of the background-only fit to the diphoton invariant mass, $m_{\gamma \gamma }$, distribution in the one lepton and two photons signal and validation regions. The contributions from SM Higgs boson production are constrained to the MC prediction and associated systematic uncertainties. The band shows the systematic uncertainty on the fit. The fit is performed on events with 100 GeV $< m_{\gamma \gamma }<$ 160 GeV, with events in SR$\ell \gamma \gamma$-1 or SR$\ell \gamma \gamma$-2 in the Higgs-mass window (120 GeV $\le m_{\gamma \gamma }\le$ 130 GeV), indicated by the *arrows*, excluded from the fit. **a** SR$\ell \gamma \gamma$-1, **b** SR$\ell \gamma \gamma$-2, **c** VR$\ell \gamma \gamma$-1, **d** VR$\ell \gamma \gamma$-2

In this channel, a neural network algorithm, based on the momenta of the tracks associated with each vertex and the direction of flight of the photons, is used to select the primary vertex, similarly to the ATLAS SM $h\rightarrow \gamma \gamma$ analysis described in Ref. [81]. Signal muons must satisfy $|d_0|<1$ mm and $|z_0|<10$ mm. The isolation criteria for both the electrons and muons are $E_\mathrm {T}^{\mathrm {cone}0.4}/p_\mathrm {T}<0.2$ and $p_\mathrm {T}^{\mathrm {cone}0.2}/p_\mathrm {T}<0.15$. Signal photons are required to satisfy $E_\mathrm {T}^{\mathrm {cone}0.4}<6{\mathrm {\ GeV}}$ and $p_\mathrm {T}^{\mathrm {cone}0.2}<2.6{\mathrm {\ GeV}}$.

The two largest background contributions are due to multi-jet and $Z\gamma$ production, with leptons or jets misreconstructed as photons. These background contributions are suppressed by requiring $E_{\mathrm {T}}^{\mathrm {miss}}>40{\mathrm {\ GeV}}$.

The $\mathbf {p}_\mathrm {T}$ of the $W\rightarrow \ell \nu$ system, reconstructed assuming background events with neutrino $\mathbf {p}_\mathrm {T}=\mathbf {p}_\mathrm {T}^\mathrm {\,miss}$, is required to be back-to-back with the $\mathbf {p}_\mathrm {T}$ of the $h\rightarrow \gamma \gamma$ candidate ($\Delta \phi (W,h)>2.25$). Only events with a diphoton invariant mass, $m_{\gamma \gamma }$, between 100 and 160 GeV are considered. Events in the sideband, outside the Higgs-mass window between 120 and 130 GeV, are included to constrain the non-Higgs background as described in Sect. 6.2.

Selected events are split into two SRs with different expected signal sensitivities based on two variables $m_{\mathrm {T}}^{{W\!\gamma _1}}$ and $m_{\mathrm {T}}^{{W\!\gamma _2}}$, which are defined as

3 $$\begin{aligned} m_\mathrm {T}^{{W\!\gamma _i}} = \sqrt{ (m_\mathrm {T}^W)^2 + 2 E_\mathrm {T}^W E_\mathrm {T}^{\gamma _i} - 2 \mathbf {p}_\mathrm {T}^W\cdot \mathbf {p}_\mathrm {T}^{\gamma _i}}, \end{aligned}$$

where $m_\mathrm {T}^W$, $E_\mathrm {T}^W$ and $\mathbf {p}_\mathrm {T}^W$ are the transverse mass, energy and momentum of the $W$ candidate, and $E_\mathrm {T}^{\gamma _i}$ and $\mathbf {p}_\mathrm {T}^{\gamma _i}$ are the transverse energy and momentum of the $i$th, $p_\mathrm {T}$-ordered, photon. Including a photon in the transverse mass calculation provides a means to identify leptonically decaying $W$ bosons in the presence of a final-state radiation photon. Events with $m_{\mathrm {T}}^{{W\!\gamma _1}}>150{\mathrm {\ GeV}}$ and $m_{\mathrm {T}}^{{W\!\gamma _2}}>80{\mathrm {\ GeV}}$ are classified into SR$\ell \gamma \gamma$-1, and those with either $m_{\mathrm {T}}^{{W\!\gamma _1}}<150{\mathrm {\ GeV}}$ or $m_{\mathrm {T}}^{{W\!\gamma _2}}<80{\mathrm {\ GeV}}$ into SR$\ell \gamma \gamma$-2. Most of the sensitivity to the signal is provided by SR$\ell \gamma \gamma$-1, while SR$\ell \gamma \gamma$-2 assists in constraining systematic uncertainties.

Two overlapping validation regions are defined by inverting and modifying the $E_{\mathrm {T}}^{\mathrm {miss}}$ and $\Delta \phi (W,h)$ criteria relative to those of the signal regions. The first region VR$\ell \gamma \gamma$-1 requires $E_{\mathrm {T}}^{\mathrm {miss}}<40{\mathrm {\ GeV}}$ and has no requirement on $\Delta \phi (W,h)$, and the second region VR$\ell \gamma \gamma$-2 requires $\Delta \phi (W,h)<2.25$ and has no requirement on $E_{\mathrm {T}}^{\mathrm {miss}}$. The signal and validation regions are summarised in Table 5.

**Table 6:** Event yields and SM expectation in the Higgs-mass window of the lepton plus two photon channel ($120<m_{\gamma \gamma }<130{\mathrm {\ GeV}}$) after the background-only fit. The Higgs-mass window is excluded from the fit in the two signal regions. The errors shown include statistical and systematic uncertainties

	SR$\ell \gamma \gamma$-1	SR$\ell \gamma \gamma$-2	VR$\ell \gamma \gamma$-1	VR$\ell \gamma \gamma$-2
Observed events	1	5	30	26
SM expectation	1.6 $\pm$ 0.4	3.3 $\pm$ 0.8	30.2 $\pm$ 2.3	20.4 $\pm$ 1.9
Non-Higgs	0.6 $\pm$ 0.3	3.0 $\pm$ 0.8	29.2 $\pm$ 2.3	19.8 $\pm$ 1.9
$Wh$	0.85 $\pm$ 0.02	0.23 $\pm$ 0.01	0.71 $\pm$ 0.02	0.29 $\pm$ 0.01
$Zh$	0.04 $\pm$ 0.01	0.02 $\pm$ 0.01	0.14 $\pm$ 0.02	0.05 $\pm$ 0.01
$t\bar{t}h$	0.14 $\pm$ 0.01	0.02 $\pm$ 0.01	0.11 $\pm$ 0.01	0.25 $\pm$ 0.01

Distributions in the Higgs-mass window of the four kinematic variables used to define the SRs are shown in Fig. 3. For illustration purposes, the observed yield in the sideband region is shown for each distribution, scaled into the corresponding Higgs-mass window by the relative widths of the Higgs-mass window and the sideband region, 10 GeV/50 GeV $=$ 0.2. Also shown, for each distribution, is a simulation-based cross-check of the background estimate. To reduce statistical uncertainties originating from the limited number of simulated events, the non-Higgs contributions are obtained in the sideband and scaled into the Higgs-mass window by 0.2. The simulation-based prediction of the non-Higgs background is estimated from the W/Z($\gamma ,\gamma \gamma$) $+$ jets samples, after applying a data-driven correction for the probability of electrons or jets to be reconstructed as photons. The contribution from backgrounds with jets reconstructed as leptons is determined by using the “fake factor” method described in Ref. [82]. This simulation-based background estimate is only used as a cross-check of the sideband-data-based background estimate described above. It gives results consistent with the data estimate, but it is not used for limit setting.

### Background estimation

The contribution from background sources that do not contain a $h\rightarrow \gamma \gamma$ decay can be statistically separated by a template fit to the full $m_{\gamma \gamma }$ distribution, from 100 to 160 GeV. The approach followed is similar to the one in Ref. [81]: the non-Higgs background is modelled as $\exp (-\alpha m_{\gamma \gamma })$, with the constant $\alpha$ as a free, positive parameter in the fit. Alternative functional models are used to evaluate the systematic uncertainty due to the choice of background modelling function. The $h\rightarrow \gamma \gamma$ template, used for the Higgs background and signal, is formed by the sum of a Crystal Ball function [83] for the core of the distribution and a Gaussian function for the tails. This functional form follows the one used in the SM $h\rightarrow \gamma \gamma$ analysis [81], with the nominal values and uncertainties on the fit parameters determined by fits to the simulation in SR$\ell \gamma \gamma$-1 and SR$\ell \gamma \gamma$-2. The results of the fit to the simulation are used as an external constraint on the template during the fit to data. The width of the Gaussian core of the Crystal Ball function quantifies the detector resolution and is determined in simulation to be 1.7 GeV in SR$\ell \gamma \gamma$-1 and 1.8 GeV in SR$\ell \gamma \gamma$-2. This is comparable to the resolution found in the SM $h\rightarrow \gamma \gamma$ analysis [81].

Contributions from SM processes with a real Higgs boson decay are estimated by simulation and come primarily from $Wh$ associated production, with smaller amounts from $t\bar{t}h$ and $Zh$. The contributions from SM Higgs boson production via gluon fusion or vector boson fusion are found to be negligible. Systematic uncertainties on the yields of these SM processes are discussed in Sect. 8. Figure 4 shows the background-only fits to the observed $m_{\gamma \gamma }$ distributions in the signal and validation regions, with the signal region Higgs-mass window ($120<m_{\gamma \gamma }<130{\mathrm {\ GeV}}$) excluded from the fit. Table 6 summarises the observed event yields in the Higgs-mass window and the background estimates, from the background-only fits, in the signal and validation regions. The errors are dominated by the statistical uncertainty due to the number of events in the $m_{\gamma \gamma }$ sidebands.

## Same-sign dilepton channel

### Event selection

Events recorded with a combination of dilepton triggers are used in the same-sign dilepton channel. The $p_\mathrm {T}$ thresholds of the dilepton triggers depend on the flavour of the leptons. The triggers reach their maximum efficiency at $p_\mathrm {T}$ values of about $14$–$25$ GeV for the leading lepton and $8$–$14$ GeV for the sub-leading lepton.

**Table 7:** Selection requirements for the signal regions of the same-sign dilepton channel

	SR$ee$-1	SR$ee$-2	SR$\mu \mu$-1	SR$\mu \mu$-2	SR$e\mu$-1	SR$e\mu$-2
Lepton flavours	$ee$	$ee$	$\mu \mu$	$\mu \mu$	$e\mu$	$e\mu$
$n_\mathrm {jet}$	1	2 or 3	1	2 or 3	1	2 or 3
Leading lepton $p_\mathrm {T}$ (GeV)	$>$30	$>$30	$>$30	$>$30	$>$30	$>$30
Sub-leading lepton $p_\mathrm {T}$ (GeV)	$>$20	$>$20	$>$20	$>$30	$>$30	$>$30
$|m_{\ell \ell }-m_Z|$ (GeV)	$>$10	$>$10	–	–	–	–
$\Delta \eta _{\ell \ell }$	–	–	$<$1.5	$<$1.5	$<$1.5	$<$1.5
$E_{\mathrm {T}}^{\mathrm {miss,rel}}$ (GeV)	$>$55	$>$30	–	–	–	–
$m_\mathrm {eff}$ (GeV)	$>$200	–	$>$200	$>$200	$>$200	$>$200
$m_\mathrm {T}^\mathrm {max}$ (GeV)	–	$>$110	$>$110	–	$>$110	$>$110
$m_{\ell j}$ or $m_{\ell jj}$ (GeV)	$<$90	$<$120	$<$90	$<$120	$<$90	$<$120

The offline event selection requires two same-sign signal leptons ($$ee$$, $$e\mu $$ or $$\mu \mu $$) with $$p_\mathrm {T}>30{\mathrm {\ GeV}}$$ or $$20{\mathrm {\ GeV}}$$ as shown in Table 7 and no additional preselected lepton. The signal electrons must satisfy the “tight” identification criteria from Ref. [66], $$|d_0|/\sigma _{d_0}<3$$, and $$|z_0\sin \theta {}|<0.4$$ mm. The signal muons must satisfy $$|\eta |<2.4$$, $$|d_0|/\sigma _{d_0}<3$$, and $$|z_0\sin \theta {}|<1$$ mm. The isolation criteria for electrons (muons) are $$E_\mathrm {T}^{\mathrm {cone}0.3}/$$$$\min (p_\mathrm {T},60{\mathrm {\ GeV}})$$$$<0.13$$ (0.14) and $$p_\mathrm {T}^{\mathrm {cone}0.3}/$$$$\min (p_\mathrm {T},60{\mathrm {\ GeV}})$$$$<0.07$$ (0.06). Events containing a hadronically decaying preselected $$\tau $$ lepton are rejected in order to avoid statistical overlap with the three-lepton final states [21].

Events are required to contain one, two, or three central ($|\eta |<2.4$) jets with $p_\mathrm {T}>20{\mathrm {\ GeV}}$. If a central jet has $p_\mathrm {T}{}<50{\mathrm {\ GeV}}$ and has tracks associated to it, at least one of the tracks must originate from the event primary vertex. To reduce background contributions with heavy-flavour decays, all the jets must fail to meet the $b$-tagging criterion at the 80 % efficiency operating point. There must be no forward ($2.4<|\eta |<4.9$) jet with $p_\mathrm {T}>30{\mathrm {\ GeV}}$.

The dominant background contributions in the $\ell ^{\pm }\ell ^{\pm }$ channel are due to SM diboson production ($WZ$ and $ZZ$) leading to two “prompt” leptons and due to events with “non-prompt” leptons (heavy-flavour decays, photon conversions and misidentified jets). These background contributions are suppressed with the tight identification criteria described above, and with the kinematic requirements summarised in Table 7. The requirements were optimised separately for each lepton flavour combination ($ee$, $\mu \mu$, and $e\mu$), and for different numbers of reconstructed jets, leading to six signal regions.

The dilepton invariant mass $m_{\ell \ell }$ is required to differ by at least 10 GeV from the $Z$-boson mass for the $ee$ channel, in which contamination due to electron charge misidentification is significant.

The visible mass of the Higgs boson candidate is defined for the one jet signal regions as the invariant mass ($m_{\ell j}$) of the jet and the lepton that is closest to it in terms of $\Delta R$, and for the two or three jet signal regions as the invariant mass ($m_{\ell jj}$) of the two highest-$p_\mathrm {T}$ jets and the lepton that is closest to the dijet system. In the signal regions, $m_{\ell j}{}<90$ GeV is required for SR$\ell \ell$-1 and $m_{\ell jj}{}<120$ GeV for SR$\ell \ell$-2.

**Fig. 5:**
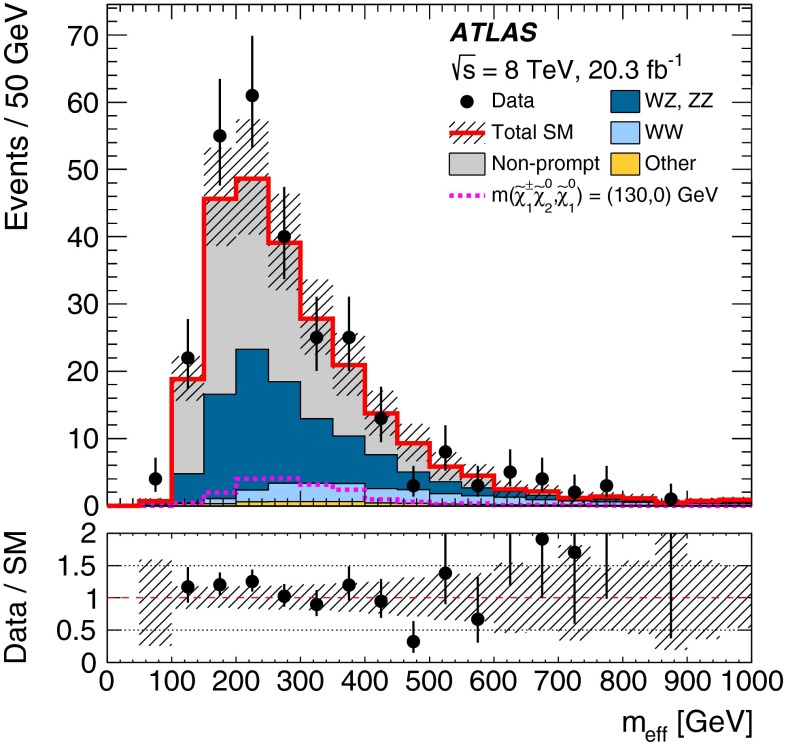
Distribution of effective mass $m_\mathrm {eff}$ in the validation region of the same-sign $e\mu$ channel. This validation region is defined by requiring one, two, or three jets, and reversing the $m_{\ell j}$, $m_{\ell jj}$ criteria. The *hashed areas* represent the total uncertainties on the background estimates that are depicted with *stacked histograms*. The distribution of a signal hypothesis is also shown *without stacking* on the background histograms. The *lower panel* shows the ratio of the data to the SM background prediction

**Fig. 6:**
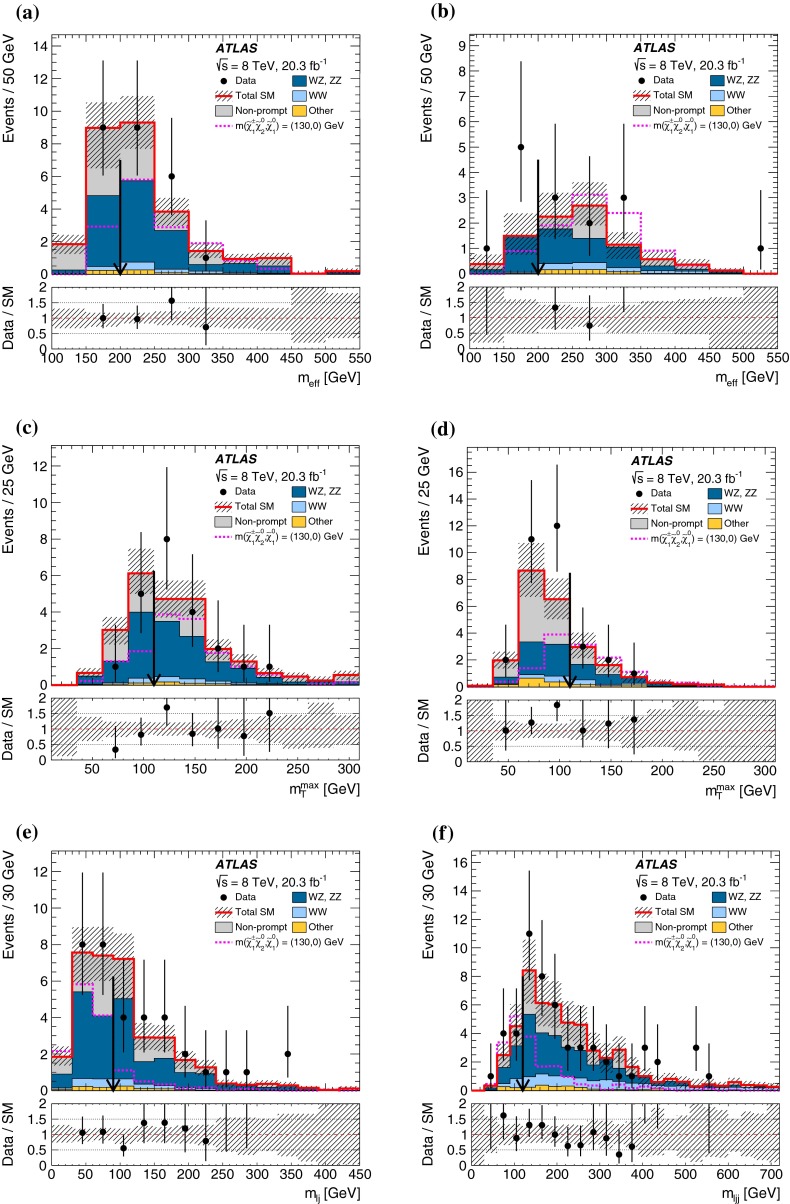
Distributions of effective mass $m_\mathrm {eff}$, largest transverse mass $m_\mathrm {T}^\mathrm {max}$, invariant mass of lepton and jets $m_{\ell j}$ and $m_{\ell jj}$ for the same-sign dilepton channel in the signal regions with one jet (*left*) and two or three jets (*right*). SR$\ell \ell$-1 is the sum of SR$ee$-1, SR$e\mu$-1, and SR$\mu \mu$-1; SR$\ell \ell$-2 is the sum of SR$ee$-2, SR$e\mu$-2, and SR$\mu \mu$-2. All selection criteria are applied, except for the one on the variable being shown. The *vertical arrows* indicate the boundaries of the signal regions, which may not apply to all flavour channels. The *hashed areas* represent the total uncertainties on the background estimates that are depicted with *stacked histograms*. The distributions of a signal hypothesis are also shown *without stacking* on the background histograms. The *lower panels* show the ratio between data and the SM background prediction. The rightmost bins of each distribution include overflow. **a** $m_\mathrm {eff}$ in SR$\ell \ell$-1 without $m_\mathrm {eff}$ cut, **b** $m_\mathrm {eff}$ in SR$\ell \ell$-2 without $m_\mathrm {eff}$ cut, **c** $m_\mathrm {T}^\mathrm {max}$ in SR$\ell \ell$-1 without $m_\mathrm {T}^\mathrm {max}$ cut, **d** $m_\mathrm {T}^\mathrm {max}$ in SR$\ell \ell$-2 without $m_\mathrm {T}^\mathrm {max}$ cut, **e** $m_{\ell j}$ in SR$\ell \ell$-1 without $m_{\ell j}$ cut, **f** $m_{\ell jj}$ in SR$\ell \ell$-2 without $m_{\ell jj}$ cut

**Table 8:** Event yields and SM expectation in the same-sign dilepton channel signal regions. The $WW$ background includes both $W^{\pm }W^{\pm }$ and $W^{\pm }W^{\mp }$ production, the latter due to electron charge mis-measurement. “Other” background includes $t\bar{t}$, single top, $Z+\mathrm {jets}$, $Zh$ and $Wh$ production. The errors shown include statistical and systematic uncertainties

	SR$ee$-1	SR$ee$-2	SR$\mu \mu$-1	SR$\mu \mu$-2	SR$e\mu$-1	SR$e\mu$-2
Observed events	2	1	6	4	8	4
SM expectation	6.0 $\pm$ 1.2	2.8 $\pm$ 0.8	3.8 $\pm$ 0.9	2.6 $\pm$ 1.1	7.0 $\pm$ 1.3	1.9 $\pm$ 0.7
Non-prompt	3.4 $\pm$ 1.0	1.6 $\pm$ 0.5	0.00 $\pm$ 0.20	0.3 $\pm$ 0.4	3.0 $\pm$ 0.9	0.48 $\pm$ 0.28
$WZ$, $ZZ$	2.2 $\pm$ 0.6	0.7 $\pm$ 0.4	3.4 $\pm$ 0.8	1.8 $\pm$ 0.9	3.3 $\pm$ 0.8	1.1 $\pm$ 0.5
$WW$	0.33 $\pm$ 0.31	0.22 $\pm$ 0.23	0.24 $\pm$ 0.29	0.4 $\pm$ 0.5	0.4 $\pm$ 0.4	0.23 $\pm$ 0.26
Other	0.13 $\pm$ 0.13	0.31 $\pm$ 0.31	0.14 $\pm$ 0.14	0.06 $\pm$ 0.06	0.19 $\pm$ 0.17	0.09 $\pm$ 0.08

Depending on the final state, additional kinematic variables are used to further reduce the background. Requiring the pseudorapidity difference between the two leptons $\Delta \eta _{\ell \ell }<1.5$ decreases the $WZ$ and $ZZ$ background. Requirements on $E_{\mathrm {T}}^{\mathrm {miss,rel}}$, defined as

4 $$\begin{aligned} E_{\mathrm {T}}^{\mathrm {miss,rel}}= \left\{ \begin{array}{ll} E_{\mathrm {T}}^{\mathrm {miss}}&{}\quad \mathrm {if}\,\,\ \Delta \phi >\pi /2, \\ E_{\mathrm {T}}^{\mathrm {miss}}\sin (\Delta \phi ) &{} \quad \mathrm {if}\,\,\ \Delta \phi <\pi /2, \\ \end{array} \right. \end{aligned}$$

where $$\Delta \phi $$ is the azimuthal angle difference between $$\mathbf {p}_\mathrm {T}^\mathrm {miss}$$ and the nearest lepton or jet, reduce the $$Z$$$$+$$ jets and non-prompt lepton background in the $$ee$$ channel. The $$E_{\mathrm {T}}^{\mathrm {miss,rel}}$$ is defined so as to reduce the impact on $$E_{\mathrm {T}}^{\mathrm {miss}}$$ of any potential mismeasurement, either from jets or from leptons. The scalar sum $$m_\mathrm {eff}$$ of the transverse momenta of the leptons, jets and the missing transverse momentum is used to suppress the diboson background. Requiring $$m_\mathrm {T}^\mathrm {max}>110{\mathrm {\ GeV}}$$, where $$m_\mathrm {T}^\mathrm {max}$$ is the larger of the two $$m_\mathrm {T}^W$$ values computed with one of the leptons and the missing transverse momentum, suppresses background events with one leptonically decaying $$W$$ boson, whose transverse mass distribution has an endpoint at $$m_W$$.

To test the non-prompt lepton and charge mismeasurement backgrounds, validation regions are defined by applying only the number of jets $n_\mathrm {jet}$ and lepton $p_\mathrm {T}$ requirements from Table 7 and requiring $m_{\ell j}>90{\mathrm {\ GeV}}$ or $m_{\ell jj}>120{\mathrm {\ GeV}}$.

### Background estimation

The irreducible background in the same-sign dilepton channel is dominated by $WZ$ and $ZZ$ diboson production, in which both vector bosons decay leptonically and one or two leptons do not satisfy the selection requirements, mostly the kinematic ones. These contributions are estimated from the simulation.

Background contributions due to non-prompt leptons are estimated with the matrix method described in Ref. [22]. It takes advantage of the difference between the efficiencies for prompt and non-prompt leptons, defined as the fractions of prompt and non-prompt preselected leptons respectively, that pass the signal-lepton requirements. The number of events containing non-prompt leptons is obtained from these efficiencies and the observed number of events using four categories of selection with preselected or signal leptons. The efficiencies for prompt and non-prompt leptons are derived, as a function of $p_\mathrm {T}$ and $\eta$, for each process leading to either prompt or non-prompt leptons using the generator-level information from simulated events. They are then corrected for potential differences between simulation and data with correction factors measured in control regions, as described in Ref. [22]. The contributions from each process leading to either prompt or non-prompt leptons are then used to compute a weighted-average efficiency, where the weight for each process is determined as its relative contribution to the number of preselected leptons in the region of interest.

Same-sign background events where the lepton charge is mismeasured are usually due to a hard bremsstrahlung photon with subsequent asymmetric pair production. The charge mismeasurement probability, which is negligible for muons, is measured in data as a function of electron $p_\mathrm {T}$ and $|\eta |$ using $Z\rightarrow e^+e^-$ events where the two electrons are reconstructed with the same charge. The probability, which is below $1\,\%$ for most of the $p_\mathrm {T}$ and $\eta$ values, is then applied to the simulated opposite-sign $ee$ and $e\mu$ pairs to estimate this background [84]. Although any process with the $e^{\pm }e^{\mp }$ or $e^{\pm }\mu ^{\mp }$ final state can mimic the same-sign signature with charge mismeasurement, most of this background contribution is due to the production of $Z+\mathrm {jets}$ events, amounting to less than $10\,\%$ of the background yield in each of the $\ell ^{\pm }\ell ^{\pm }$ signal regions.

Estimates of non-prompt lepton and charge mismeasurement background are tested in the validation regions; the number of observed events agrees with the expected background in all validation regions. Figure 5 shows the distribution of $m_\mathrm {eff}$ in the validation region of the same-sign $e\mu$ channel.

The number of observed and expected events in each signal region is reported in Table 8. Figure 6 shows the data distributions of $m_\mathrm {eff}$, $m_\mathrm {T}^\mathrm {max}$, $m_{\ell j}$, and $m_{\ell jj}$ compared to the SM expectations in the same-sign dilepton signal regions. No significant excess is observed over the SM background expectations in any channel.

## Systematic uncertainties

**Table 9:** Summary of the statistical and main systematic uncertainties on the background estimates, expressed in per cent of the total background yields in each signal region. Uncertainties that are not considered for a particular channel are indicated by a “–”. The individual uncertainties can be correlated, and do not necessarily add in quadrature to the total background uncertainty

	SR$\ell bb$-1	SR$\ell bb$-2	SR$\ell \gamma \gamma$-1	SR$\ell \gamma \gamma$-2	SR$\ell \ell$-1	SR$\ell \ell$-2
Number of background events	$6.0\pm 1.3$	$2.8\pm 0.8$	$1.6\pm 0.4$	$3.3\pm 0.8$	$16.8\pm 2.8$	$7.3~\pm ~1.5$
Statistical	9	7	22	23	7	7
Modelling $t\bar{t}$	23	25	–	–	–	–
Modelling single top	5	11	–	–	–	–
Modelling $Wh$, $Zh$, $t\bar{t}h$	–	–	3	1	–	–
Modelling $WZ$	–	–	–	–	11	22
Electron reconstruction	3	3	1	1	$<$1	$<$1
Muon reconstruction	1	1	$<$1	$<$1	1	$<$1
Photon reconstruction	–	–	4	5	–	–
Jet energy scale and resolution	6	14	1	3	2	11
$b$-jet identification	6	4	–	–	–	–
$m_{bb}$ shape	8	12	–	–	–	–
Background $m_{\gamma \gamma }$ model	–	–	5	7	–	–
Non-prompt estimate	–	–	–	–	10	11
Charge mismeasurement estimate	–	–	–	–	2	3
Other sources	4	5	$<$1	2	2	2

Table 9 summarises the dominant systematic uncertainties on the total expected background yields in the six signal regions.

For the one lepton and two $b$-jets channel, theoretical uncertainties on the $t\bar{t}$ and single-top background estimates are the most important. They are evaluated by comparing different generators (Powheg, MC@NLO  [85, 86] and AcerMC) and parton shower algorithms (Pythia6 and Herwig  [87, 88]), varying the QCD factorisation and renormalisation scales up and down by a factor of two, and taking the envelope of the background variations when using different PDF sets. Statistical uncertainties from the data in the CRs result in uncertainties on the normalisations of the $t\bar{t}$ and $W+\mathrm {jets}$ backgrounds, while the limited number of simulated events yields uncertainty on the shape of the background $m_{bb}$ distributions. The largest experimental systematic uncertainties are those on the jet energy scale [72] and resolution [89], derived from a combination of test-beam data and in-situ measurements, followed by the uncertainty on the $b$-jet identification efficiency [90]. The uncertainty on the $W$ boson background modelling is dominated by the uncertainty on the cross section for the production of the $W$ boson in association with heavy-flavour jets, and is reported within the “Other sources”. The $W$ boson background component is small in $\ell {}bb$ SRs, and its uncertainty is constrained by the CRs with a similar composition.

For the one lepton and two photons channel, the background uncertainties are dominated by the data statistics in the $m_{\gamma \gamma }$ sidebands. The only source of systematic uncertainty on the non-Higgs background estimate is the choice of $m_{\gamma \gamma }$ model. The systematic uncertainties on the Higgs background estimates are dominated by the theoretical uncertainties on the $Wh$, $Zh$, and $t\bar{t}h$ production cross sections and the photon reconstruction. The main theoretical uncertainties are those on the QCD scales and the parton distribution functions [55]. The effect of scale uncertainties on the modelling of Higgs boson production is evaluated by reweighting the simulated Higgs boson $p_\mathrm {T}$ distribution to account for doubling and halving the scales. The experimental systematic uncertainty from photon reconstruction is determined with the tag-and-probe method using radiative $Z$ decays [91].

For the same-sign dilepton channel, the two main sources of systematic uncertainty are related to the non-prompt lepton estimate, and to the modelling of the $WZ$ background. The uncertainty on the non-prompt estimate originates mainly from the limited accuracy of the efficiency correction factors, and on the production rate of non-prompt leptons, in particular their $\eta$ dependence. The uncertainty on the $WZ$ background modelling is determined using a same-sign, $WZ$-enriched sample used to validate the Sherpa prediction. This validation sample is selected by requiring three leptons, two of which must have same flavour, opposite sign, $|m_{\ell \ell }{}-m_{Z}|<10~{\mathrm {\ GeV}}{}$, and then considering only the highest-$p_\mathrm {T}$ same-sign pair. None of the other requirements from Table 7 are applied, except for the lepton $p_\mathrm {T}$ and $n_\mathrm {jet}$ selections.

## Results and interpretations

The event yields observed in data are consistent with the Standard Model expectations within uncertainties in all signal regions. The results are used to set exclusion limits with the frequentist hypothesis test based on the profile log-likelihood-ratio test statistic and approximated with asymptotic formulae [92].

**Table 10:** From left to right, observed 95 % CL upper limits ($\langle \sigma _\mathrm{vis}\rangle _{\mathrm {obs}}^{95}$) on the visible cross sections, the observed ($S_\mathrm {obs}^{95}$) and expected ($S_\mathrm {exp}^{95}$) 95 % CL upper limits on the number of signal events with $\pm 1\sigma$ excursions of the expectation, the observed confidence level of the background-only hypothesis ($CL_B$), and the discovery $p$-value ($p_0$), truncated at $0.5$

	$\langle \sigma _\mathrm{vis}\rangle _{\mathrm {obs}}^{95}$(fb)	$S_\mathrm {obs}^{95}$	$S_\mathrm {exp}^{95}$	$CL_B$	$p_0$
SR$\ell bb$-1	0.26	5.3	${6.3}^{+3.4}_{-2.0}$	0.28	0.50
SR$\ell bb$-2	0.27	5.5	${5.1}^{+2.6}_{-1.4}$	0.56	0.43
SR$\ell \gamma \gamma$-1	0.18	3.6	${4.1}^{+2.0}_{-0.7}$	0.25	0.50
SR$\ell \gamma \gamma$-2	0.34	7.0	${5.9}^{+2.0}_{-1.2}$	0.75	0.19
SR$\ell \ell$-1	0.51	10.4	$10.9^{+3.8}_{-3.1}$	0.51	0.50
SR$\ell \ell$-2	0.51	10.3	$8.1^{+3.3}_{-1.5}$	0.72	0.32

**Fig. 7:**
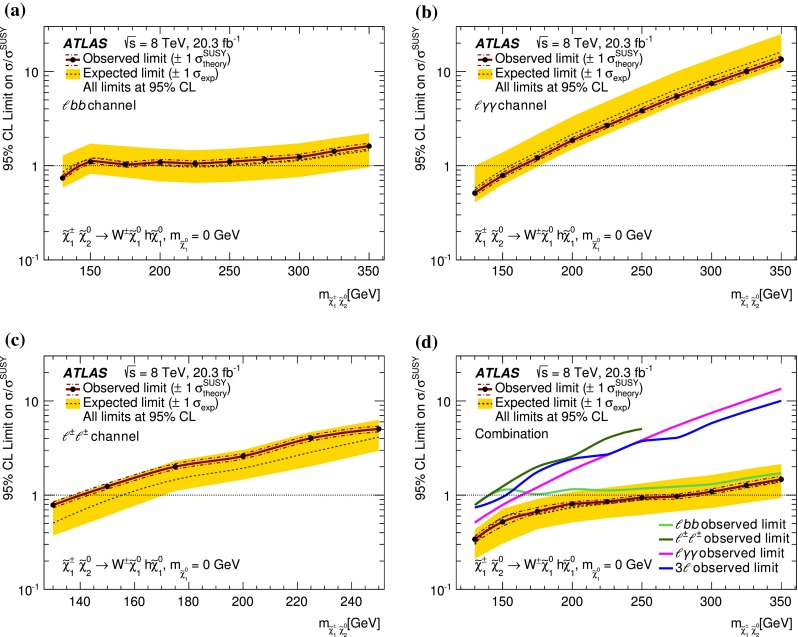
Observed (*solid line*) and expected (*dashed line*) 95 % CL upper limits on the cross section normalised by the simplified model prediction as a function of the common mass $m_{\tilde{\chi }_1^\pm \tilde{\chi }_2^0}$ for $m_{{\tilde{\chi }_1^0}}=0$. The combination in **d** is obtained using the result from the ATLAS three-lepton search [21] in addition to the three channels reported in this paper. The *dash-dotted lines* around the observed limit represent the results obtained when changing the nominal signal cross section up or down by the $\pm 1\sigma _{\text {theory}}^{\text {SUSY}}$ theoretical uncertainty. The *solid band around the expected limit* represents the $\pm 1\sigma _{\text {exp}}$ uncertainty band where all uncertainties, except those on the signal cross sections, are considered. **a** One lepton and two $b$-jets channel, **b** one lepton and two photons channel, **c** same-sign dilepton channel, **d** combination

**Fig. 8:**
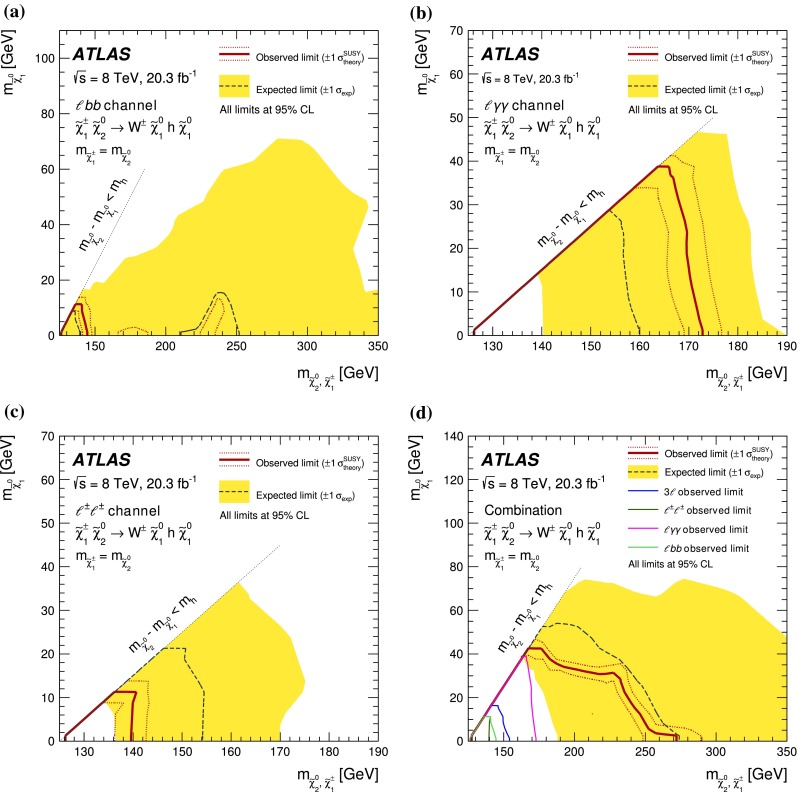
Observed (*solid line*) and expected (*dashed line*) 95 % CL exclusion regions in the mass plane of $m_{\tilde{\chi }_1^0}$ vs.  $m_{\tilde{\chi }_2^0,\tilde{\chi }_1^\pm }$ in the simplified model. The combination in **d** is obtained using the result from the ATLAS three-lepton search [21] in addition to the three channels reported in this paper. The *dotted lines around the observed limit* represent the results obtained when changing the nominal signal cross section up or down by the $\pm 1 \sigma _{\text {theory}}^{\text {SUSY}}$ theoretical uncertainty. The *solid band around the expected limit* shows the $\pm 1\sigma _{\text {exp}}$ uncertainty band where all uncertainties, except those on the signal cross sections, are considered. **a** One lepton and two $b$-jets channel, **b** one lepton and two photons channel, **c** same-sign dilepton channel and **d** combination

Exclusion upper limits at the 95 % confidence level (CL) on the number of beyond-the-SM (BSM) signal events, $S$, for each SR are derived using the CL$_\mathrm {s}$ prescription [93], assuming no signal yield in other signal and control regions. Normalising the upper limits on the number of signal events by the integrated luminosity of the data sample provides upper limits on the visible BSM cross section, $\sigma _\mathrm{vis} = \sigma \times A \times \epsilon$, where $\sigma$ is the production cross section for the BSM signal, $A$ is the acceptance defined as the fraction of events passing the geometric and kinematic selections at particle level, and $\epsilon$ is the detector reconstruction, identification and trigger efficiency.

Table 10 summarises, for each SR, the observed 95 % CL upper limits ($\langle \sigma _\mathrm{vis}\rangle _{\mathrm {obs}}^{95}$) on the visible cross section, the observed ($S_\mathrm {obs}^{95}$) and expected ($S_\mathrm {exp}^{95}$) 95 % CL upper limits on the number of signal events with $\pm 1\sigma$ excursions of the expectation, the observed confidence level ($CL_B$) of the background-only hypothesis, and the discovery $p$-value ($p_0$), truncated at $0.5$.

The results are also used to set exclusion limits on the common mass of the $\tilde{\chi }_1^\pm$ and $\tilde{\chi }_2^0$ for various values of the $\tilde{\chi }_1^0$ mass in the simplified model of $pp\rightarrow \tilde{\chi }_1^\pm \tilde{\chi }_2^0$ followed by $\tilde{\chi }_1^\pm \rightarrow W^\pm \tilde{\chi }_1^0$ and $\tilde{\chi }_2^0\rightarrow h\tilde{\chi }_1^0$. In this hypothesis test, all the CRs and SRs, including the data in the Higgs-mass windows of the $\ell {}bb$ and $\ell \gamma \gamma$ channels, are fitted simultaneously, taking into account correlated experimental and theoretical systematic uncertainties as common nuisance parameters. The signal contamination in the CRs is accounted for in the fit, where a single non-negative normalisation parameter is used to describe the signal model in all channels.

Systematic uncertainties on the signal expectations stemming from detector effects are included in the fit in the same way as for the backgrounds. Theoretical systematic uncertainties on the signal cross section described in Sect. 3 are not included directly in the fit. In all resulting exclusions the dashed (black) and solid (red) lines show the 95 % CL expected and observed limits respectively, including all uncertainties except for the theoretical signal cross-section uncertainty. The (yellow) bands around the expected limit show the $\pm 1\sigma _{\text {exp}}$ expectations. The dotted $\pm 1\sigma _{\text {theory}}^{\text {SUSY}}$ (red) lines around the observed limit represent the results obtained when changing the nominal signal cross section up or down by its theoretical uncertainty, and reported limits correspond to the $-1\sigma {}$ variation.

Figure 7 shows the 95 % CL upper limits on the signal cross section normalised by the simplified-model prediction as a function of $m_{\tilde{\chi }_2^0,\tilde{\chi }_1^\pm }$ for $m_{{\tilde{\chi }_1^0}}=0$. The sensitivity of the individual one lepton and two $b$-jets, one lepton and two photons, and same-sign dilepton channels is illustrated in Fig. 7a–c respectively. The corresponding limit combining all channels and the ATLAS three-lepton search is shown in Fig. 7d. For $m_{\tilde{\chi }_2^0,\tilde{\chi }_1^\pm }>250$ GeV the same-sign dilepton channel is not considered. In Fig. 7a, the expected exclusion region below $m_{\tilde{\chi }_2^0,\tilde{\chi }_1^\pm }=140$ GeV is largely due to SR$\ell bb$-1, which targets models with small mass splitting between the neutralinos, while the expected exclusion region around $m_{\tilde{\chi }_2^0,\tilde{\chi }_1^\pm }=240$ GeV is driven by SR$\ell bb$-2 designed for larger mass splittings. The upper limit shows slow variation with increasing $m_{\tilde{\chi }_2^0,\tilde{\chi }_1^\pm }$ as the acceptance of SR$\ell bb$-2 increases and compensates for the decrease of the production cross section. Figure 7d shows that in the $m_{\tilde{\chi }_2^0,\tilde{\chi }_1^\pm }<170$ GeV range all channels show similar sensitivity, while for $m_{\tilde{\chi }_2^0,\tilde{\chi }_1^\pm }>170$ GeV the one lepton and two $b$-jets channel is the dominant one. Nevertheless, the contribution from the other channels to the combination is important to extend the excluded range significantly compared to Fig. 7a.

Figure 8a–c show the 95 % CL exclusion regions in the $(m_{\tilde{\chi }_2^0,\tilde{\chi }_1^\pm }, m_{\tilde{\chi }_1^0})$ mass plane of the simplified model obtained from the individual one lepton and two $b$-jets, one lepton and two photons, and same-sign dilepton signal regions, respectively. Figure 8d shows the corresponding exclusion region obtained by combining the three channels described in this paper with the ATLAS three-lepton search, which by itself excludes $m_{\tilde{\chi }_2^0{}, \tilde{\chi }_1^\pm {}}$ up to 160 GeV for $m_{\tilde{\chi }_1^0}=0$ as seen in Fig. 8d. The combination of these four independent searches improves the sensitivity significantly, and the 95 % CL exclusion region for $m_{{\tilde{\chi }_1^0}}=0$ is extended to 250 GeV. The wide uncertainty bands of the expected limits in Fig. 8 are due to the slow variation of the sensitivity with increasing $m_{\tilde{\chi }_2^0,\tilde{\chi }_1^\pm }$ and $m_{\tilde{\chi }_1^0}$, as can also be seen in Fig. 7. In a similar search by the CMS Collaboration [25], the observed limit on $m_{\tilde{\chi }_2^0{}, \tilde{\chi }_1^\pm {}}$ is 210 GeV for $m_{{\tilde{\chi }_1^0}}=0$.

## Conclusions

A search for the direct pair production of a chargino and a neutralino $pp\rightarrow \tilde{\chi }_1^\pm \tilde{\chi }_2^0$ followed by $\tilde{\chi }^\pm \rightarrow \tilde{\chi }_1^0(W^{\pm }\rightarrow \ell ^{\pm }\nu )$ and $\tilde{\chi }_2^0\rightarrow \tilde{\chi }_1^0(h\rightarrow bb/\gamma \gamma /\ell ^{\pm }\nu qq)$ has been performed using 20.3 $\mathrm {fb}^{-1}$ of $\sqrt{s}=8{\mathrm {\ TeV}}$ proton–proton collision data delivered by the Large Hadron Collider and recorded with the ATLAS detector. Three final-state signatures are considered: one lepton and two $b$-jets, one lepton and two photons, and two same-sign leptons, each associated with missing transverse momentum. Observations are consistent with the Standard Model expectations. Limits are set in a simplified model, combining these results with the three-lepton search presented in Ref. [21]. For the simplified model, common masses of $\tilde{\chi }_1^\pm$ and $\tilde{\chi }_2^0$ are excluded up to 250 GeV for a massless $\tilde{\chi }_1^0$.

## Footnotes

1 ATLAS uses a right-handed coordinate system with its origin at the nominal interaction point (IP) in the centre of the detector and the $z$-axis along the beam line. The $x$-axis points from the IP to the centre of the LHC ring, and the $y$-axis points upward. Cylindrical coordinates $(r,\phi )$ are used in the transverse plane, $\phi$ being the azimuthal angle around the $z$-axis. The pseudorapidity is defined in terms of the polar angle $\theta$ as $\eta =-\ln \tan (\theta /2)$.
